# The metabolic profile of the synthetic cannabinoid receptor agonist ADB-HEXINACA using human hepatocytes, LC–QTOF-MS and synthesized reference standards

**DOI:** 10.1093/jat/bkad065

**Published:** 2023-09-25

**Authors:** Steven R Baginski, Tobias Rautio, Lorna A Nisbet, Karin Lindbom, Xiongyu Wu, Johan Dahlén, Craig McKenzie, Henrik Gréen

**Affiliations:** Leverhulme Research Centre for Forensic Science, School of Science and Engineering, University of Dundee, Fleming Laboratory, Small’s Wynd, Dundee DD1 4HN, UK; Department of Physics, Chemistry and Biology, Linköping University, Linköping 581 83, Sweden; Leverhulme Research Centre for Forensic Science, School of Science and Engineering, University of Dundee, Fleming Laboratory, Small’s Wynd, Dundee DD1 4HN, UK; Division of Clinical Chemistry and Pharmacology, Department of Biomedical and Clinical Sciences, Faculty of Medicine and Health Sciences, Linköping University, Linköping 581 83, Sweden; Department of Physics, Chemistry and Biology, Linköping University, Linköping 581 83, Sweden; Department of Physics, Chemistry and Biology, Linköping University, Linköping 581 83, Sweden; Leverhulme Research Centre for Forensic Science, School of Science and Engineering, University of Dundee, Fleming Laboratory, Small’s Wynd, Dundee DD1 4HN, UK; Chiron AS, Stiklestadveien 1, Trondheim 7041, Norway; Division of Clinical Chemistry and Pharmacology, Department of Biomedical and Clinical Sciences, Faculty of Medicine and Health Sciences, Linköping University, Linköping 581 83, Sweden; Department of Forensic Genetics and Forensic Toxicology, National Board of Forensic Medicine, Artillerigatan 12, Linköping 587 58, Sweden

## Abstract

Synthetic cannabinoid receptor agonists (SCRAs) remain a major public health concern, with their use implicated in intoxications and drug-related deaths worldwide. Increasing our systematic understanding of SCRA metabolism supports clinical and forensic toxicology casework, facilitating the timely identification of analytical targets for toxicological screening procedures and confirmatory analysis. This is particularly important as new SCRAs continue to emerge on the illicit drug market. In this work, the metabolism of ADB-HEXINACA (ADB-HINACA, *N*-[1-amino-3,3-dimethyl-1-oxobutan-2-yl]-1-hexyl-1*H*-indazole-3-carboxamide), which has increased in prevalence in the United Kingdom and other jurisdictions, was investigated using *in vitro* techniques. The (*S*)-enantiomer of ADB-HEXINACA was incubated with pooled human hepatocytes over 3 hours to identify unique and abundant metabolites using liquid chromatography–quadrupole time-of-flight mass spectrometry. In total, 16 metabolites were identified, resulting from mono-hydroxylation, di-hydroxylation, ketone formation (mono-hydroxylation then dehydrogenation), carboxylic acid formation, terminal amide hydrolysis, dihydrodiol formation, glucuronidation and combinations thereof. The majority of metabolism took place on the hexyl tail, forming ketone and mono-hydroxylated products. The major metabolite was the 5-oxo-hexyl product (M9), while the most significant mono-hydroxylation product was the 4-hydroxy-hexyl product (M8), both of which were confirmed by comparison to in-house synthesized reference standards. The 5-hydroxy-hexyl (M6) and 6-hydroxy-hexyl (M7) metabolites were not chromatographically resolved, and the 5-hydroxy-hexyl product was the second largest mono-hydroxylated metabolite. The structures of the terminal amide hydrolysis products without (M16, third largest metabolite) and with the 5-positioned ketone (M13) were also confirmed by comparison to synthesized reference standards, along with the 4-oxo-hexyl metabolite (M11). The 5-oxo-hexyl and 4-hydroxy-hexyl metabolites are suggested as biomarkers for ADB-HEXINACA consumption.

## Introduction

Synthetic cannabinoid receptor agonists (SCRAs) are a major public health concern, with their use implicated in intoxications and drug-related deaths worldwide, particularly in vulnerable prison and homeless populations (1, 2). SCRAs are novel or new psychoactive substances (NPS) designed to mimic the effects of ∆^9^-tetrahydrocannabinol (∆^9^-THC), the main psychoactive component of cannabis. These compounds bind to and activate the cannabinoid receptors, CB_1_ and CB_2_, resulting in a number of pharmacological effects such as euphoria and disinhibition (1–3). However, numerous negative effects have been reported, including seizures, reduced consciousness, anxiety, aggression, tachycardia, heart attack, cardiac arrest, respiratory depression and death (1, 2, 4). Activation of CB_1_ is primarily responsible for the psychoactive effects, while CB_2_ activation is linked to anti-inflammatory responses, and unlike ∆^9^-THC, a partial agonist of cannabinoid receptors, SCRAs are full agonists (1–3). SCRAs on the illicit drug market are constantly evolving, often because producers create new compounds to circumvent newly introduced national legislation (particularly in producer countries) and international legislation. More than 224 SCRAs have been reported to the European Monitoring Centre for Drugs and Drug Addiction (EMCDDA), and this group of compounds has become the largest to be monitored by this agency (5). During the last 2 years the number of emerging SCRAs has increased significantly, with 15 new compounds reported to the EMCDDA in 2021 and more than 20 new SCRAs in 2022, compared to an average of around 10 per year from 2016 to 2020 (5).

Identification of SCRA metabolites is crucial for clinical and forensic toxicology casework, in order to provide analytical targets for screening procedures and confirmatory analysis as evidence of intake of parent compounds. The potency (6–9), lipophilicity (10, 11) and relatively rapid and extensive metabolism (11, 12) of most of the SCRAs studied to date means that the parent compound may only be present at low concentrations in some body fluids and tissues, particularly in urine, and may therefore be difficult to detect, making the identification of abundant and unique metabolites important for determining analytical targets to prove SCRA consumption (12, 13). Metabolites are concentrated in urine, a sample matrix that typically provides longer detection windows compared to blood, making urine an ideal sample for SCRA analysis (13). The unpredictable toxicity of SCRAs has, however, resulted in few controlled administration studies in humans; these have largely concentrated on the metabolism of earlier emerging SCRAs such as JWH-018 (14–17). Use of *in vitro* incubations with cryopreserved human hepatocytes (HHeps) is therefore often regarded as the “gold standard” technique for modeling *in vivo* metabolism. The majority of biotransformation processes take place in the liver; the major phase I and phase II enzymes, co-factors, drug transporters and drug-binding proteins involved in metabolism are therefore present in HHeps, and using multiple HHep donors can provide an average metabolism profile for the population (12). In addition to providing analytical targets, systematic *in vitro* incubations of structurally related compounds allow structure–metabolism relationships to be identified and developed, which may aid prediction of the metabolites of emerging and prophetic compounds (18).

**Figure 1.**
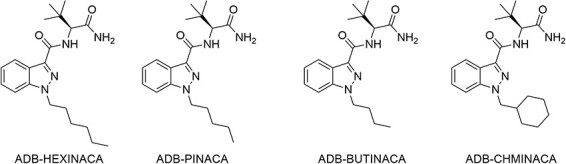
Structures of ADB-HEXINACA and structural analogs.

ADB-HEXINACA (ADB-HINACA, *N*-[1-amino-3,3-dimethyl-1oxobutan-2-yl]-1-hexyl-1*H*-indazole-3-carboxamide, Figure 1) is a *tert*-leucinamide SCRA that was first detected in the UK in April 2021, in seized herbal material in Manchester (19) and in infused papers in Scotland (20). In the USA, ADB-HEXINACA was identified in a seized herbal sample in April 2021 (21), while the first detection in blotters in Scottish prisons was reported in May 2021 (20). Since then, ADB-HEXINACA has become one of the most prevalent SCRAs in the UK and was identified in 118 drug reports recorded by the National Forensic Laboratory Information System (NFLIS) in the USA in 2021 (22). While the synthesis of ADB-HEXINACA has been detailed (19) and the metabolic profiles of structural analogs ADB-BUTINACA (ADB-BINACA, adbb, *N*-[1-amino-3,3-dimethyl-1-oxobutan-2-yl]-1-butyl-1*H*-indazole-3-carboxamide) (20, 23, 24), ADB-PINACA (*N*-[1-amino-3,3-dimethyl-1-oxobutan-2-yl]-1-pentyl-1*H*-indazole-3-carboxamide) (25) and ADB-CHMINACA (MAB-CHMINACA, *N*-[1-amino-3,3-dimethyl-1-oxobutan-2-yl]-1-(cyclohexylmethyl)-1*H*-indazole-3-carboxamide) (Figure 1) (26) have been reported, the metabolism of ADB-HEXINACA is yet to be elucidated.

In this paper, we report the biotransformation of ADB-HEXINACA using HHep incubations, followed by analysis of incubates by ultra-high performance liquid chromatography–quadrupole time-of-flight mass spectrometry (UHPLC–QTOF-MS), as well as the confirmation of the structures of some of the major metabolites using in-house synthesized reference standards. This work forms part of a wider study to systematically identify the metabolites of emerging SCRAs, to identify structure–metabolism relationships for prediction of the metabolism of future SCRAs and to support the timely availability of relevant SCRA metabolite reference standards when they are most needed.

## Materials and methods

### Materials

Williams E medium, l-glutamine, HEPES buffer and mobile phase materials (LC–MS grade acetonitrile and formic acid) were purchased from Thermo Fisher Scientific (Gothenburg, Sweden). Ethanol was from Kemetyl AB (Jordbro, Sweden). InVitro Gro HT thawing medium and cryopreserved primary HHeps (LiverPool, 20 donor pool, lot: BEK) were obtained from Bioreclamation IVT (Brussels, Belgium).

### Human hepatocyte incubation

(*S*)-ADB-HEXINACA (synthesized at Linköping University) was incubated with HHeps, and the metabolites were identified as previously described in a published procedure (27–29), but with slight modifications. In short, 5 µM of ADB-HEXINACA was incubated with HHeps (100,000 cells) in a total volume of 100 μL of cell culture media (Williams E medium supplemented with l-glutamine and HEPES). The incubations were carried out in duplicate and stopped after 0, 0.5, 1 and 3 h by the addition of 100 µL ice-cold acetonitrile. After centrifugation at 1,100 *g* for 15 min at 4°C, the supernatant was analyzed by LC–QTOF-MS. Negative controls (HHeps without drug) and degradation controls (drug without HHeps) were also incubated for 3 h.

### LC–QTOF-MS analysis

Supernatant (4 µL) was injected and separated on an Acquity HSS T3 column (150 mm × 2.1 mm, 1.8 μm; Waters, Sollentuna, Sweden) fitted with an Acquity VanGuard precolumn (Waters, Sollentuna, Sweden). LC–QTOF-MS analysis was performed on an Agilent 1290 Infinity UHPLC system (Agilent Technologies, Kista, Sweden) coupled to an Agilent 6550 iFunnel QTOF mass spectrometer (Agilent Technologies, Kista, Sweden) with a Dual Agilent Jet Stream electrospray ionization (ESI) source. Mobile phases of (A) 0.1% formic acid in water and (B) 0.1% formic acid in acetonitrile were used in gradient mode: 1% B (0–0.6 min), 1–20% B (0.6–0.7 min), 20–85% B (0.7–13 min), 85–95% B (13–15 min), 95% B (15–18 min), 95–1% B (18–18.1 min) and 1% B (18.1–19 min). The flow rate was 0.5 mL/min and the column temperature was 60°C. Mass spectrometric data were acquired in positive ESI mode using Auto MS-MS acquisition with the following parameters: scan range, *m/z* 100–950 (MS) and *m/z* 50–950 (MS-MS); precursor intensity threshold, 5,000 counts; precursor number per cycle, 5; fragmentor voltage, 380 V; collision energy (CE), 3 eV at *m/z* 0 ramped up by 8 eV per *m/z* 100; gas temperature, 150°C; gas flow, 18 L/min; nebulizer gas pressure, 345 kPa; sheath gas temperature, 375°C and sheath gas flow, 11 L/min.

### Metabolite identification

Data analysis was performed with Agilent MassHunter Qualitative Analysis software (version B.07.00) as described previously (20). The following parameters were used: mass error, 20 ppm; absolute peak area threshold, 20,000 counts; maximum number of matches, 20 and chromatogram extraction window, 50 ppm. For metabolite identification, the following criteria had to be met: mass error <5 ppm for [M + H]^+^ (except for saturated peaks where the mass accuracy could deviate), consistent isotopic pattern, product ion spectrum consistent with the proposed structure, retention time (RT) between 2 and 13 min and plausible for the proposed structure, and absence of the peaks in the negative and degradation controls. Where a metabolite was identified in at least one sample timepoint, but present at low concentrations (below or around a peak area threshold of 20,000 counts) in other samples, a manual assessment was carried out (using automatic or manual integration with a peak area threshold of 5,000 counts) to decide whether the substance should be considered a metabolite or not. Ten predicted metabolites were synthesized (in-house at Linköping University), and the RTs and product ion spectra of these standards were compared to those of the metabolites detected in the HHep incubations.

### Synthesis of reference standards

(*S*)-ADB-HEXINACA and six oxidized products (4-hydroxy-hexyl, 4-oxo-hexyl, 5-hydroxy-hexyl, 5-oxo-hexyl, 6-hydroxy-hexyl and 6-oxo-hexyl) were synthesized. The terminal amide hydrolysis product was also synthesized, without and in combination with the 5-oxo-hexyl, 5-hydroxy-hexyl and 6-hydroxy-hexyl derivatives. Analytical characterization data for these reference standards are available in Supplementary Data S1. Peak assignment for ^1^H NMR spectra revealed no major impurities in the synthesized standards, indicating purities of >95%.

## Results and discussion

**Figure 2.**
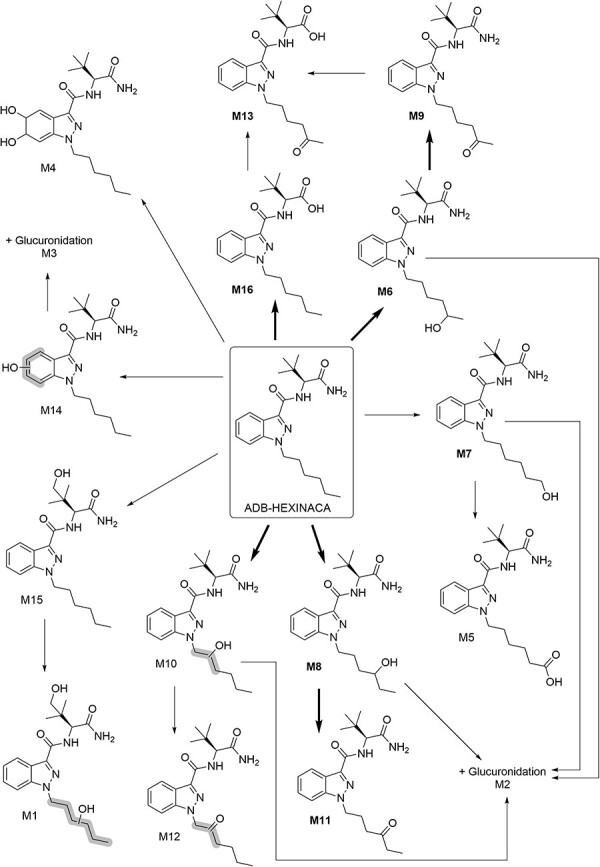
Proposed metabolic pathway of ADB-HEXINACA following incubation with human hepatocytes. Metabolites in bold font were confirmed using synthesized reference standards, and bold arrows indicate a major metabolic pathway.

**Figure 3.**
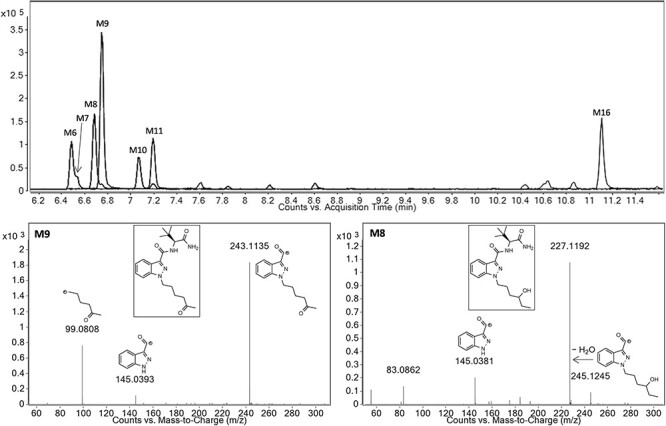
Overlaid extracted ion chromatograms of the major ADB-HEXINACA metabolites formed after 3 h incubation, and product ion spectra of the 5-oxo-hexyl (M9) and 4-hydroxy-hexyl (M8) metabolites, the two recommended biomarkers of ADB-HEXINACA consumption.

A total of 16 ADB-HEXINACA metabolites were identified in the HHep incubations, resulting from mono-hydroxylation, di-hydroxylation, ketone formation (mono-hydroxylation + dehydrogenation), carboxylic acid formation, terminal amide hydrolysis, dihydrodiol formation, glucuronidation and combinations thereof. The parent drug eluted at 10.30 min, while the metabolites eluted between 5.21 and 11.12 min. All metabolites are listed in Table I, which details the biotransformation, chemical formula, accurate mass of the protonated molecule, mass error (maximum and minimum), RT, peak area and rank (based on average peak area at 3 h). The proposed metabolic pathway of ADB-HEXINACA is depicted in Figure 2, while extracted ion chromatograms and mass spectra are shown in Figure 3 and Supplementary Data S2.

**Table I.** ADB-HEXINACA Metabolites with Biotransformation, Chemical Formula, Accurate Mass of the Protonated Molecule, Mass Error (Maximum and Minimum), Retention Time, Peak Area and Rank

				Mass error (ppm)			Peak area (× 1000)						
Met ID	Biotransformation	Chemical formula	[M + H]^+^ (*m/z*)	Max	Min	Mean RT (min)	0.5 h—A	0.5 h—B	1 h—A	1 h—B	3 h—A	3 h—B	Rank
	ADB-HEXINACA	C_20_H_30_N_4_O_2_	359.2467	7.92	4.08	10.30	11,718	11,939	7,689	7,706	2,910	3,763	–
M1	Di-hydroxylation (hexyl tail + *tert*-butyl)	C_20_H_30_N_4_O_4_	391.2344	1.87	−0.30	5.21	22	*17*	19	23	48	44	10
M2	Mono-hydroxylation + glucuronidation (hexyl tail)	C_26_H_38_N_4_O_9_	551.2723	2.82	2.17	5.21	*13*	*8*	21	*22*	60	56	9
M3	Mono-hydroxylation + glucuronidation (indazole ring)	C_26_H_38_N_4_O_9_	551.2722	2.86	1.33	5.71	41	30	39	46	102	109	7
M4	Dihydrodiol formation (indazole ring)	C_20_H_32_N_4_O_4_	393.2504	2.36	1.75	6.16	*16*	*13*	*17*	*20*	33	35	14
M5	Carboxylic acid formation (hexanoic acid)	C_20_H_28_N_4_O_4_	389.2190	2.74	0.15	6.31	*13*	*10*	*19*	21	78	86	8
M6/M7	Mono-hydroxylation (5-hydroxy-hexyl/6-hydroxy-hexyl)	C_20_H_30_N_4_O_3_	375.2399	2.95	1.68	6.48	236	211	198	214	248	306	4
M8	Mono-hydroxylation (4-hydroxy-hexyl)	C_20_H_30_N_4_O_3_	375.2400	2.95	1.81	6.68	369	320	297	320	360	416	2
M9	Ketone formation (5-oxo-hexyl)	C_20_H_28_N_4_O_3_	373.2245	3.60	1.08	6.75	621	565	548	648	659	793	1
M10	Mono-hydroxylation (hexyl tail)	C_20_H_30_N_4_O_3_	375.2397	2.91	1.14	7.07	171	129	129	140	149	171	6
M11	Ketone formation (4-oxo-hexyl)	C_20_H_28_N_4_O_3_	373.2243	4.41	1.99	7.20	200	191	190	218	243	278	5
M12	Ketone formation (hexyl tail)	C_20_H_28_N_4_O_3_	373.2237	4.52	−1.87	7.61	32	21	26	29	38	37	12
M13	Terminal amide hydrolysis + ketone formation (5-oxo-hexyl)	C_20_H_27_N_3_O_4_	374.2084	3.53	1.90	7.67	*9*	*8*	*11*	*12*	43	46	11
M14	Mono-hydroxylation (indazole ring)	C_20_H_30_N_4_O_3_	375.2394	2.08	−0.75	8.23	*19*	*17*	21	24	*19*	23	15
M15	Mono-hydroxylation (*tert*-butyl)	C_20_H_30_N_4_O_3_	375.2399	3.26	0.36	8.61	50	50	40	48	32	37	13
M16	Terminal amide hydrolysis	C_20_H_29_N_3_O_3_	360.2293	4.15	1.49	11.12	176	171	226	237	336	393	3

Rank: based on average peak area at 3 h. *Italics: peak areas below or around 20,000 counts, which were manually assessed.*

The majority of metabolism took place on the hexyl tail, forming phase I ketone and mono-hydroxylated products. The most abundant metabolite after 3 h was a ketone produced through oxidation reactions at the 5-position of the hexyl tail (M9, 5-oxo-hexyl, RT 6.75 min, *m/z* 373.2245), while the most abundant mono-hydroxylation (second most abundant metabolite overall) was the 4-hydroxy-hexyl metabolite (M8, RT 6.68 min, *m/z* 375.2400), both of which were confirmed with in-house synthesized standards. The 5-oxo-hexyl and 4-hydroxy-hexyl products are sufficiently unique and abundant to be recommended as urinary biomarkers of ADB-HEXINACA consumption. Ketone metabolites are not typically the most abundant metabolites for SCRAs studied to date, with hydroxylation, ester/amide hydrolysis, *N*-dealkylation, defluorination and dihydrodiol formation tending to be favored, depending on the structural features present (18, 28). However, as observed in this study, a ketone product was identified as the major metabolite for the structurally related compound ADB-PINACA, in incubations with HHeps (25). For AB-PINACA, a ketone that was the third most abundant metabolite in HHep incubations was only low-ranking in two authentic urine samples; however, a combined amide hydrolysis + ketone product *was* the major metabolite in both specimens (30). Similarly, a combined ester hydrolysis + ketone metabolite was the major urinary metabolite of EDMB-PINACA (31), and hydroxy + ketone products were some of the most abundant urinary metabolites for adamantyl SCRAs APINACA (AKB-48) and 5F-APINACA (5F-AKB-48) (32), showing that ketone formation is a major *in vivo* metabolic pathway for some compounds. Ketone metabolites have also been detected in human liver microsome (HLM) incubations of oxindole hydrazide (OXIZID) SCRAs (33, 34), which are significantly different in structure to earlier generations of SCRAs.

In addition to M8, five other mono-hydroxylated metabolites were identified. The second largest mono-hydroxylation peak (in total, the fourth most abundant) resulted from the combined 5-hydroxy-hexyl (M6) and 6-hydroxy-hexyl (M7) products (RT 6.48 min, *m/z* 375.2399), which were not chromatographically baseline-separated (Supplementary Data S2). However, the small difference in RT observed when the synthesized standards were analyzed (Supplementary Data S1) indicated that the 5-hydroxy-hexyl metabolite is the major metabolite of these two compounds. Based on previous evidence of increased retention time as the numbered position of mono-hydroxylation on a SCRA tail decreased for APINACA, and the abundances of hydroxylated APINACA metabolites in an authentic urine sample (35), M10 (RT 7.07 min, *m/z* 375.2397), the sixth most abundant ADB-HEXINACA metabolite overall, is likely to be the 3-hydroxy-hexyl metabolite. However, the structure was not confirmed with a synthesized standard nor was the suspected further oxidized ketone product derivative (M12).

Hydroxylation on sites other than the hexyl tail was less significant. Low abundances were observed for metabolites produced by mono-hydroxylation of the indazole ring (M14, RT 8.23 min, *m/z* 375.2394) and mono-hydroxylation of the *tert*-butyl moiety (M15, RT 8.61, *m/z* 375.2399), which were ranked 15th and 13th in abundance, respectively. The mono-hydroxylated indazole ring product (M14) does, however, undergo extensive phase II metabolism to form the seventh most abundant metabolite, a glucuronide (M3, RT 5.71 min, *m/z* 551.2722), thus increasing its polarity further for excretion. This is an important consideration in clinical and forensic laboratories, as β-glucuronidase is typically used in urine analysis procedures to deconjugate any glucuronides formed during phase II metabolism, in order to increase phase I metabolite or parent drug concentration and hence aid detection (12, 36). It should be noted that glucuronides are poorly ionized and undergo in-source fragmentation in ESI mass spectrometry, resulting in reduced signal (37–39). It is therefore possible that M2 (RT 5.21 min, *m/z* 551.2723), a mono-hydroxylated + glucuronidated hexyl tail product, is of higher concentration than its abundance would otherwise suggest. If M2 is hydrolyzed to the 4-hydroxy-hexyl metabolite, M8, it could result in a hydroxy-hexyl product as the most abundant phase I metabolite, in preference to the ketone, M9. A di-hydroxylated metabolite (M1, RT 5.21 min, *m/z* 391.2344), in which both the hexyl tail and *tert*-butyl moiety were hydroxylated, was also detected in the incubations but was of low abundance. This resulted in increased background noise in the product ion spectra (Supplementary Data S2), though the precursor ion and two diagnostic product ions, *m/z* 227 and 145, did corroborate the structure and matched well with the spectra of the other hydroxylated metabolites. Comparison with a synthesized reference standard would have strengthened this data, allowing structural confirmation.

As the third most abundant metabolite, terminal amide hydrolysis was also a prominent feature of ADB-HEXINACA metabolism (M16, RT 11.12 min, *m/z* 360.2293) and was confirmed with a synthesized standard. MDMB-HEXINACA, an envisioned structural analog of ADB-HEXINACA in which the terminal amide group is replaced with a methyl ester, would, however, undergo metabolism to give the same hydrolysis product. This is highly likely to occur to an even greater extent than for ADB-HEXINACA considering the greater instability of esters to hydrolysis compared to amides (18). While this analog has not yet been detected on the illicit drug market, this potentially limits the usefulness of M16 as a urinary biomarker for determining ADB-HEXINACA use. M13 (RT 7.67 min, *m/z* 374.2084), produced via a combination of terminal amide hydrolysis *and* ketone formation at the 5-position of the hexyl tail, was also confirmed with a synthesized standard, but this metabolite was far less abundant and similarly lacks uniqueness. Three of the reference standards synthesized for this study—the 6-oxo-hexyl product and the terminal amide hydrolysis + 5-hydroxy-hexyl or 6-hydroxy-hexyl products—were not detected in the incubations.

Comparison to previous metabolite identification studies enables structure–metabolism relationships to be investigated. In HHep incubations with ADB-BUTINACA, the dihydrodiol and mono-hydroxylated indazole ring metabolites were ranked first and third in terms of abundance, respectively (20), and these have been confirmed as major urinary metabolites *in vivo* (23, 24). These were the least abundant metabolites of ADB-HEXINACA (M4 and M14) and were only mid-ranked for ADB-PINACA, in which the pentyl chain was the main site of biotransformation, producing ketone and mono-hydroxylated products as the three major metabolites (25). This clearly demonstrates that greater metabolism occurs on longer SCRA tails (hexyl and pentyl) compared to those with a shorter (butyl) chain, in which biotransformation at the core is favored. For ADB-CHMINACA, extensive metabolism occurred on the cyclohexylmethyl tail; the five most abundant metabolites resulted from hydroxylations on this moiety (26). However, the only ketone product formed was a relatively minor metabolite and no glucuronides were detected. Reduced steric hindrance of acyclic alkyl chains compared to the bulkier and more rigid cyclohexylmethyl moiety may result in greater interaction with metabolizing enzymes and therefore make these biotransformations more favorable for acyclic tails.

There are some limitations to this metabolite identification study. First, while UHPLC–QTOF-MS is a very sensitive and selective technique, the different ionization efficiencies of molecules in ESI mass spectrometry mean that signal response may not accurately correspond to metabolite concentration (39), so some caution is needed in ranking the metabolites according to peak areas, particularly regarding glucuronides. Furthermore, the metabolites in the HHep incubations have not yet been confirmed in authentic clinical and forensic samples, which would strengthen the *in vitro* data. Nonetheless, use of synthesized reference standards allowed confirmation of seven of the ADB-HEXINACA metabolites in this study, including the exact site of metabolism, and *in vitro* HHep incubation data for the structural analog ADB-BUTINACA matched well with *in vivo* data from blood and urine samples (20, 24), giving added confidence that the results of this study are likely to be representative of metabolism *in vivo*.

## Conclusions

In summary, 16 metabolites of ADB-HEXINACA were identified in incubations with HHeps, with seven confirmed using synthesized reference standards. The 5-oxo-hexyl (M9) and 4-hydroxy-hexyl (M8) products are recommended as biomarkers for ADB-HEXINACA consumption. Structure–metabolism relationships have been identified for ADB-HEXINACA and its analogs, indicating greater metabolism on the tails of *tert*-leucinamide SCRAs with longer alkyl chains, and comparatively greater biotransformation on the indazole core as tail length decreases. This knowledge can be applied to future SCRAs to allow prediction of the major metabolites.

## Supplementary Material

bkad065_Supp

## Data Availability

The data for this article are available in the article and in its online supplementary material.
