# Supplementary material for: The metabolic profile of the synthetic cannabinoid receptor agonist ADB-HEXINACA using human hepatocytes, LC–QTOF-MS and synthesized reference standards
Source: J Anal Toxicol. 2023 Sep 25;47(9):826–34. doi: 10.1093/jat/bkad065 (PMC10714907; doi:10.1093/jat/bkad065)
Supplement: bkad065_Supp [file bkad065_supp.zip › jat-23-3987-File006.docx]

**The Metabolic Profile of the Synthetic Cannabinoid Receptor Agonist ADB-HEXINACA using Human Hepatocytes, LC-QTOF-MS and Synthesized Reference Standards**

Steven R. Baginski^1^, Tobias Rautio^2^, Lorna A. Nisbet^1^, Karin Lindbom^3^, Xiongyu Wu^2^, Johan Dahlen^2^, Craig McKenzie^1,4^, and Henrik Gréen^3,5^

^1^Leverhulme Research Centre for Forensic Science, School of Science and Engineering, University of Dundee, Dundee, UK

^2^Department of Physics, Chemistry and Biology, Linköping University, Linköping, Sweden

^3^Division of Clinical Chemistry and Pharmacology, Department of Biomedical and Clinical Sciences, Faculty of Medicine and Health Sciences, Linköping University, Linköping, Sweden

^4^Chiron AS, Trondheim, Norway

^5^Department of Forensic Genetics and Forensic Toxicology, National Board of Forensic Medicine, Linköping, Sweden

**Supplementary Data S1**

**Analytical Characterization Data for Synthesized Reference Standards**

**COOH-ADB-HEXINACA**

^1^H-NMR (CDCl_3_, 500 MHz) δ: 8.33 (d, *J* = 8.2 Hz, 1H), 7.57 (d, *J* = 9.6 Hz, 1H), 7.42-7.24 (m, 2H), 7.27-7.24 (m, 1H), 4.76 (d, *J* = 9.6 Hz, 1H), 4.39 (t, *J* = 7.2 Hz, 1H), 1.91-1.97 (m, 2H), 1.26-1.37 (m, 6H), 1.14 (s, 9H), 0.87 (t, *J* = 7.0 Hz, 3H). ^13^C-NMR (CDCl_3_, 126 MHz) δ: 176.2, 162.7, 140.9, 136.5, 126.8, 123.0, 122.9, 122.8, 109.4, 59.8, 49.6, 35.1, 31.4, 29.8, 26.8, 26.6, 22.6, 14.1.


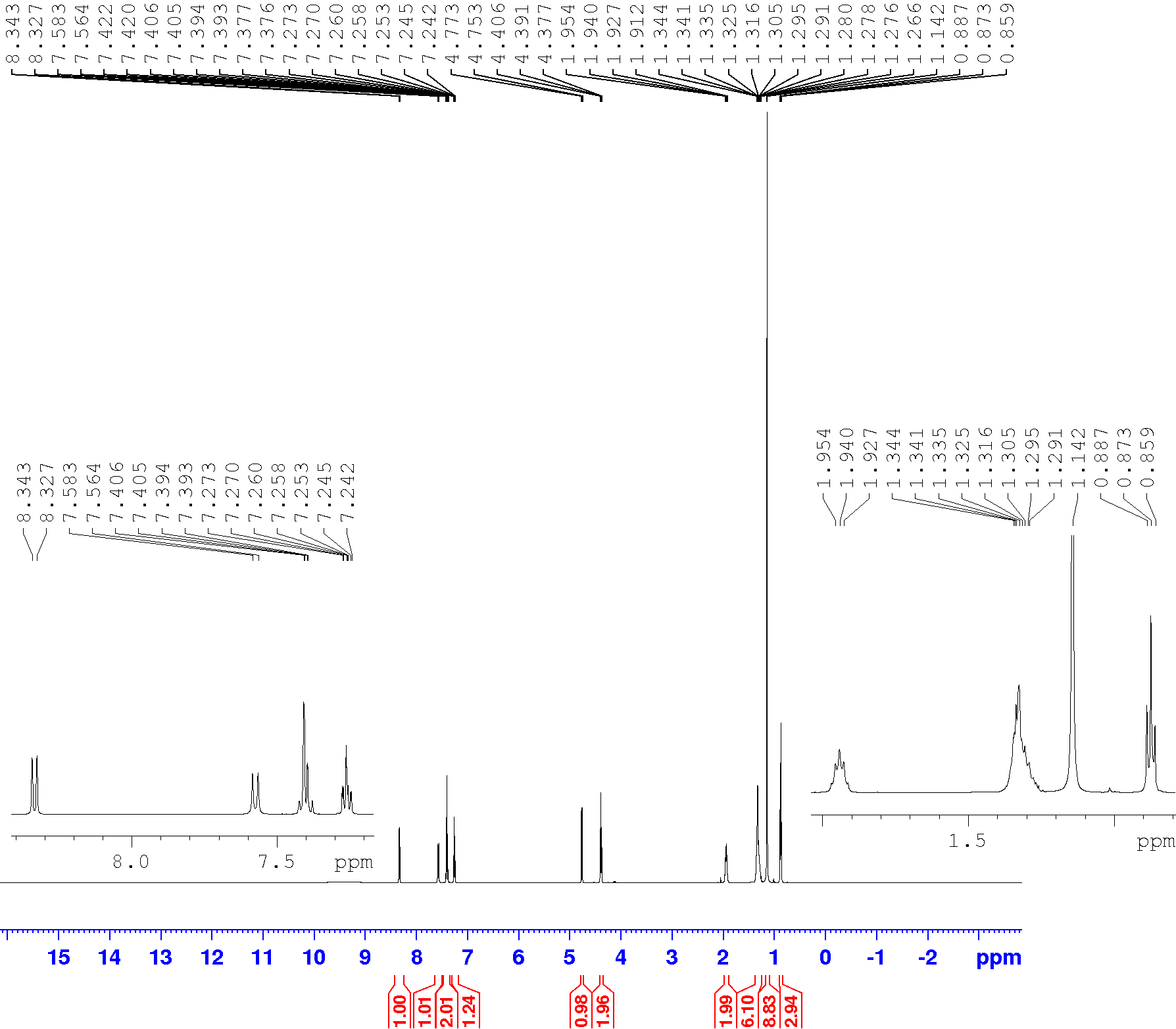


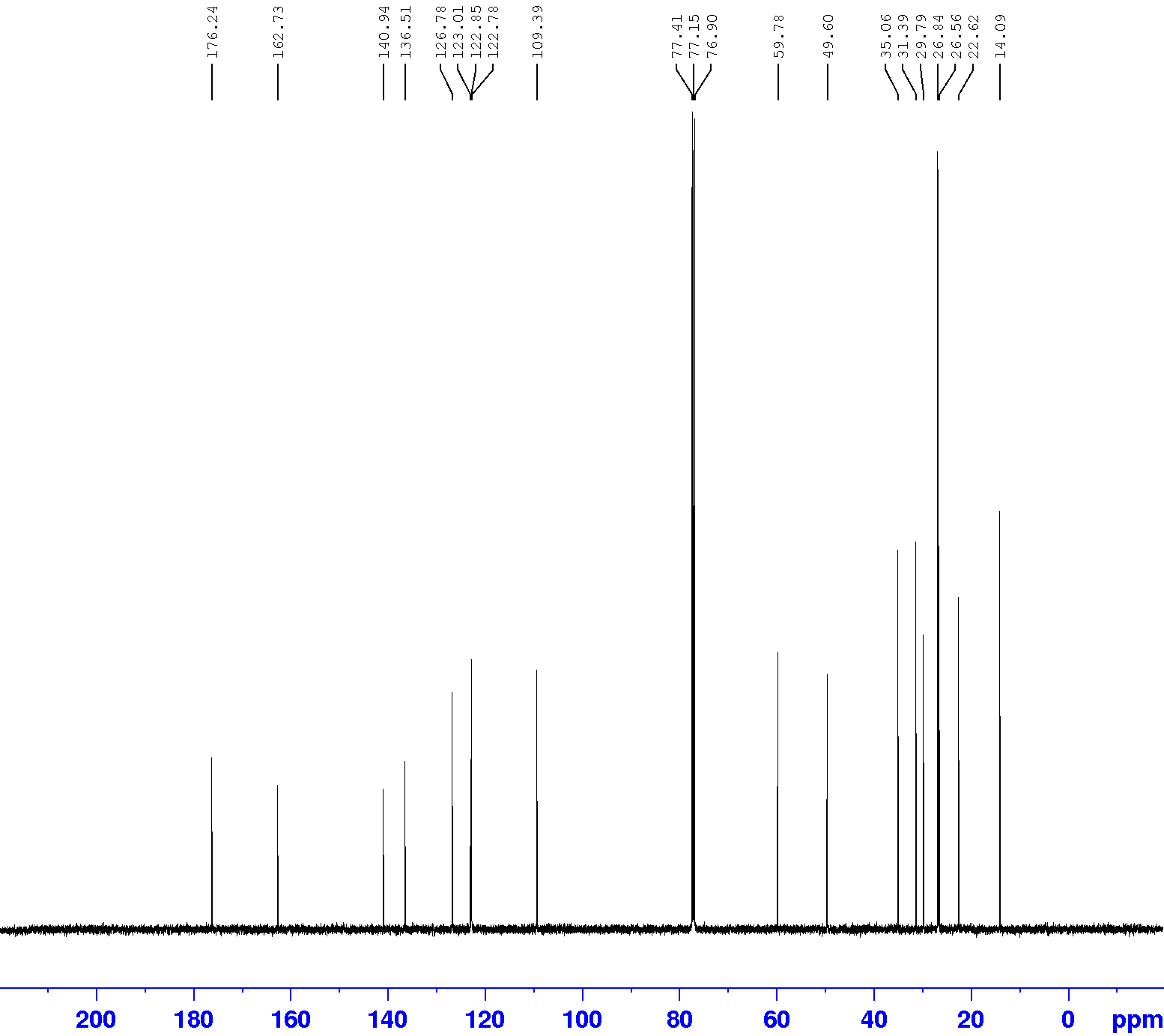

**6OH-ADB-HEXINACA**

^1^H-NMR (CDCl_3_, 500 MHz) δ: 8.25 (d, *J* = 8.2 Hz, 1H), 7.75 (d, *J* = 9.7 Hz, 1H), 7.36-7.40 (m, 2H), 7.21-7.24 (m, 1H), 6.99 (bs, 1H), 6.10 (bs, 1H), 4.72 (d, *J* = 9.7 Hz, 1H), 4.38 (t, *J* = 6.9 Hz, 2H), 3.60 (t, *J* = 6.4 Hz, 2H), 2.54 (bs, 1H), 1.93 (quin, *J* = 7.1 Hz, 2H), 1..43-1.59 (m, 3H), 1.23-1.41 (m, 3H), 1.14 (s, 9H). ^13^C-NMR (CDCl_3_, 126 MHz) δ: 173.4, 162.8, 140.9, 136.5, 126.7, 123.0, 122.7, 122.5, 109.5, 62.5, 59.7, 49.1, 34.8, 32.5, 29.4, 26.9, 26.8, 26.3, 25.0.


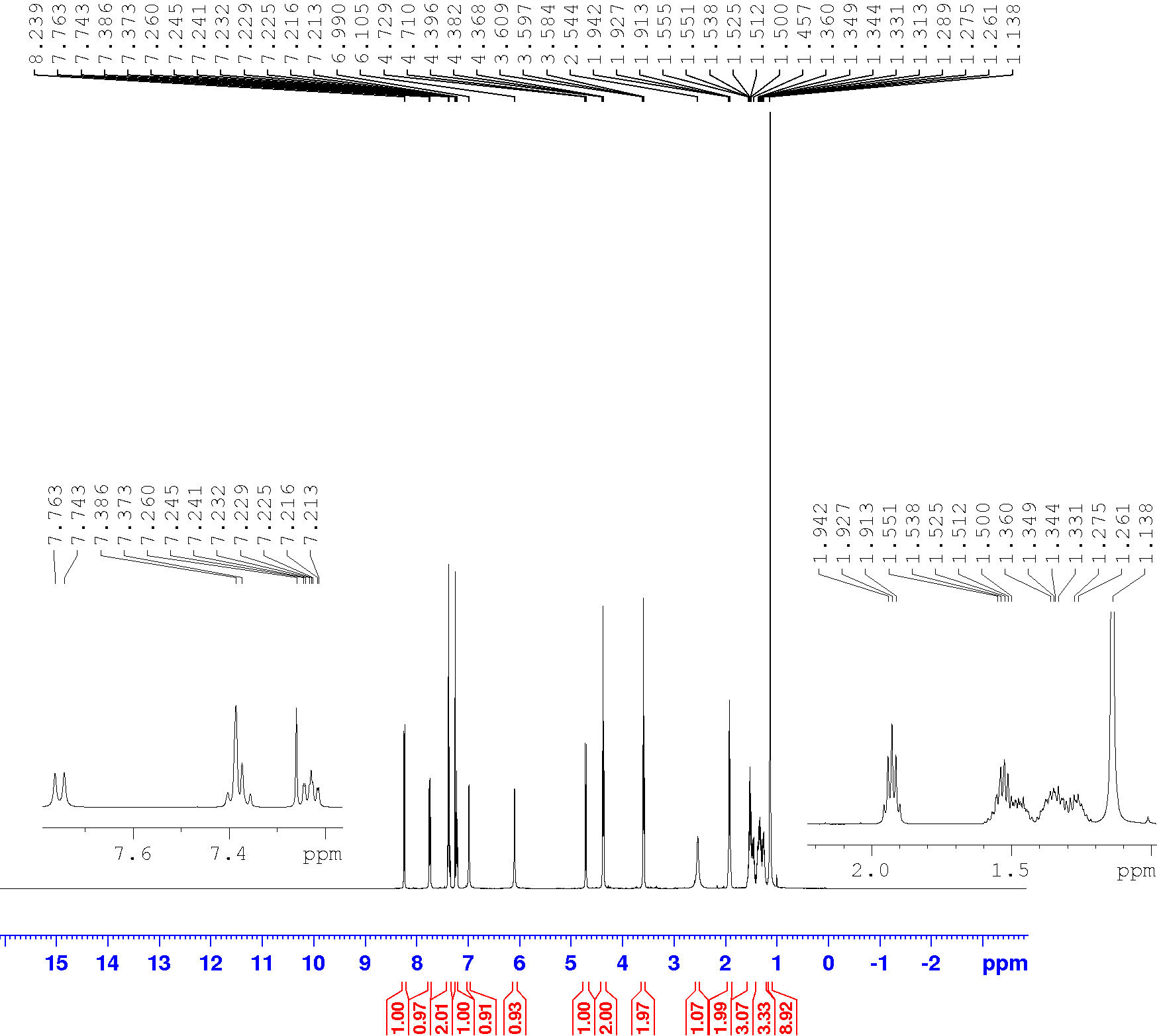


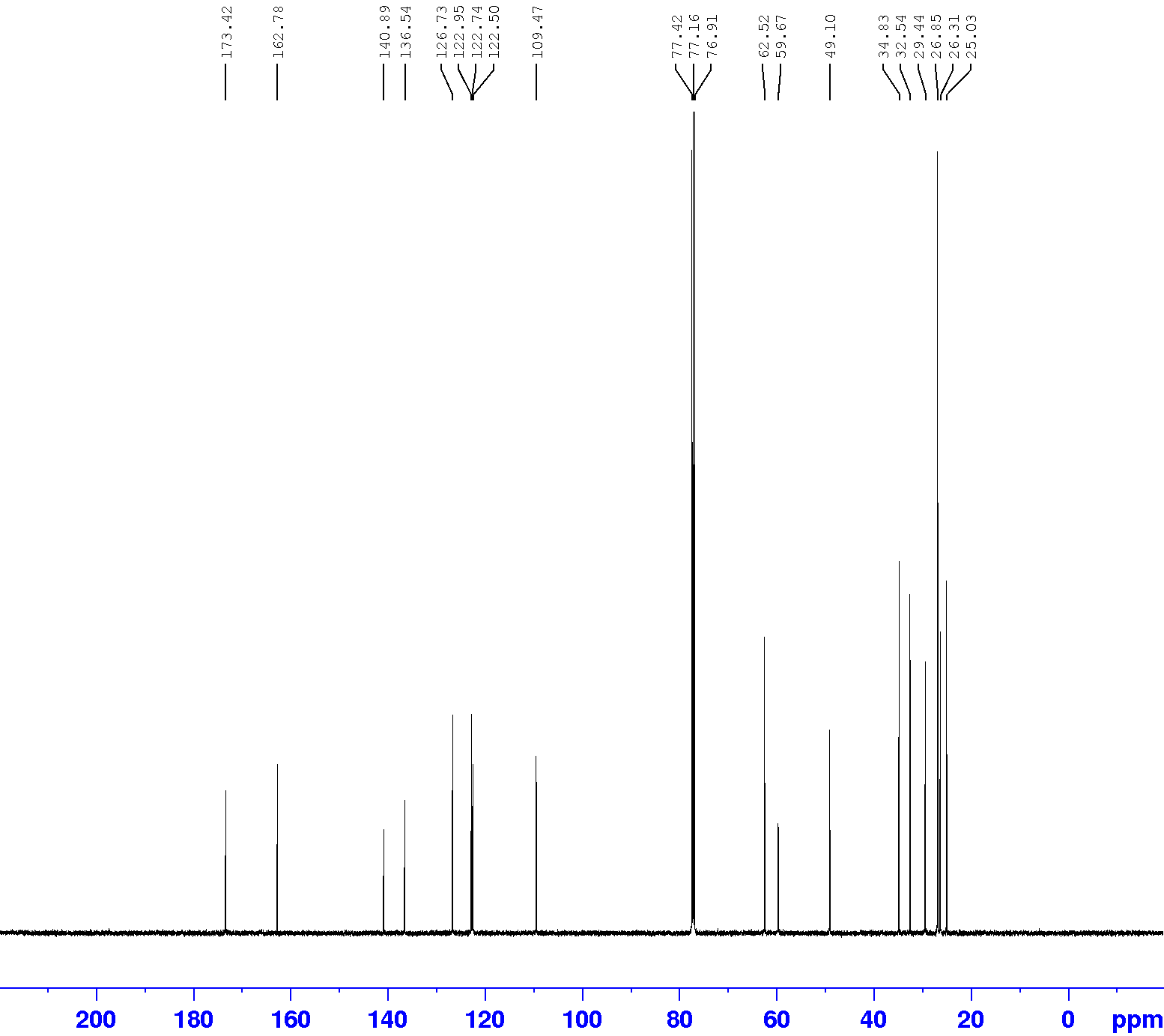

**6OH-COOH-ADB-HEXINACA**

^1^H-NMR (CDCl_3_, 500 MHz) δ: 8.31 (d, *J* = 8.2 Hz, 1H), 7.63 (d, *J* = 9.6 Hz, 1H), 7.36-7.37 (m, 2H), 7.22-7.26 (m, 1H), 6.09 (bs, 1H), 4.70 (d, *J* = 9.6 Hz, 1H), 4.37 (t, *J* = 7.2 Hz, 1H), 3.60 (t, *J* = 7.2 Hz, 1H), 1.88-1.95 (m, 2H), 1.51-1.54 (m, 2H), 1.20-1.47 (m, 4H) 1.11 (s, 9H). ^13^C-NMR (CDCl_3_, 126 MHz) δ: 175.0, 162.7, 140.9, 136.6, 126.8, 123.0, 122.9, 122.8, 109.4, 62.6, 59.9, 49.0, 35.0, 32.3, 29.3, 26.9, 26.3, 24.9.


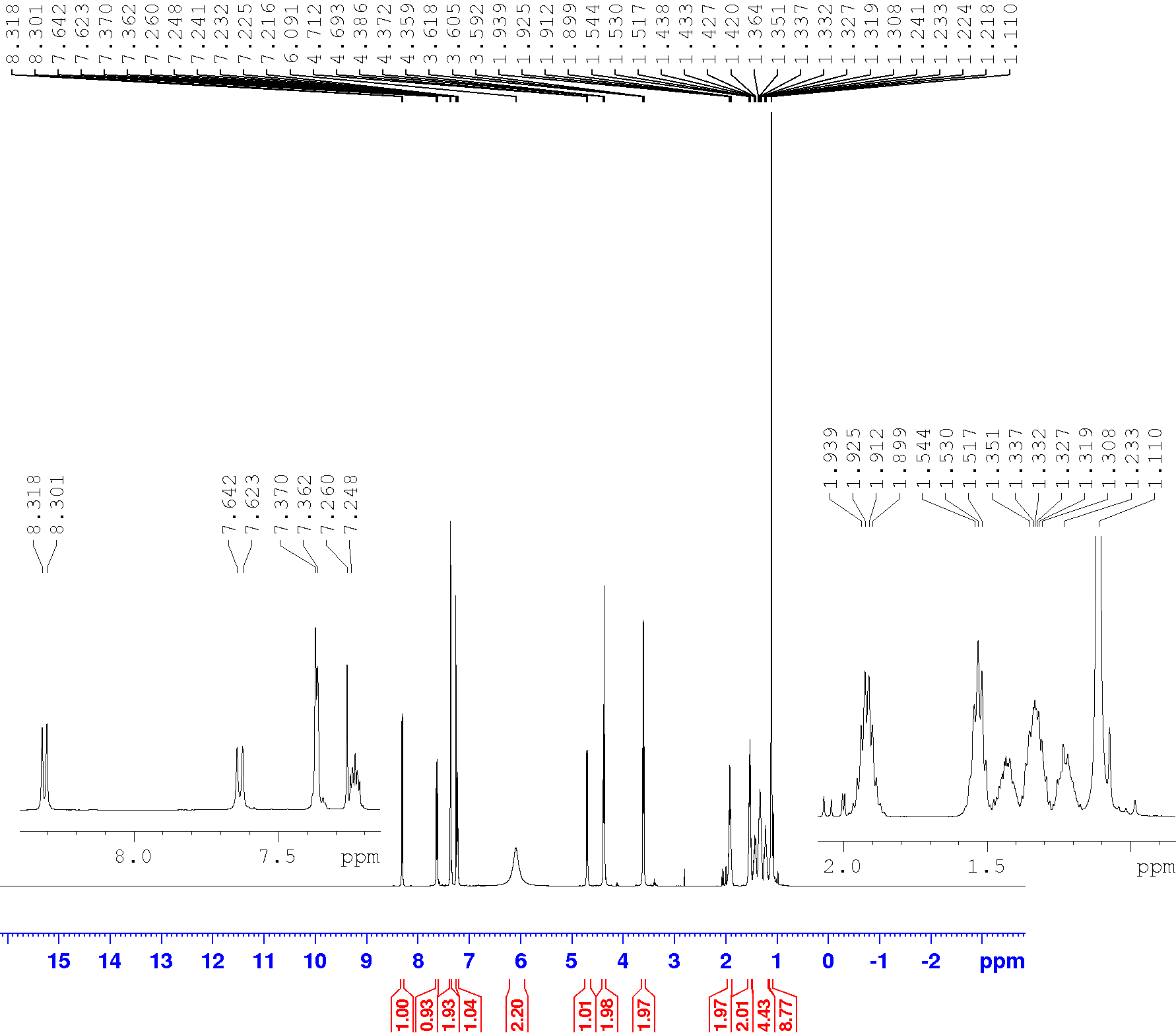


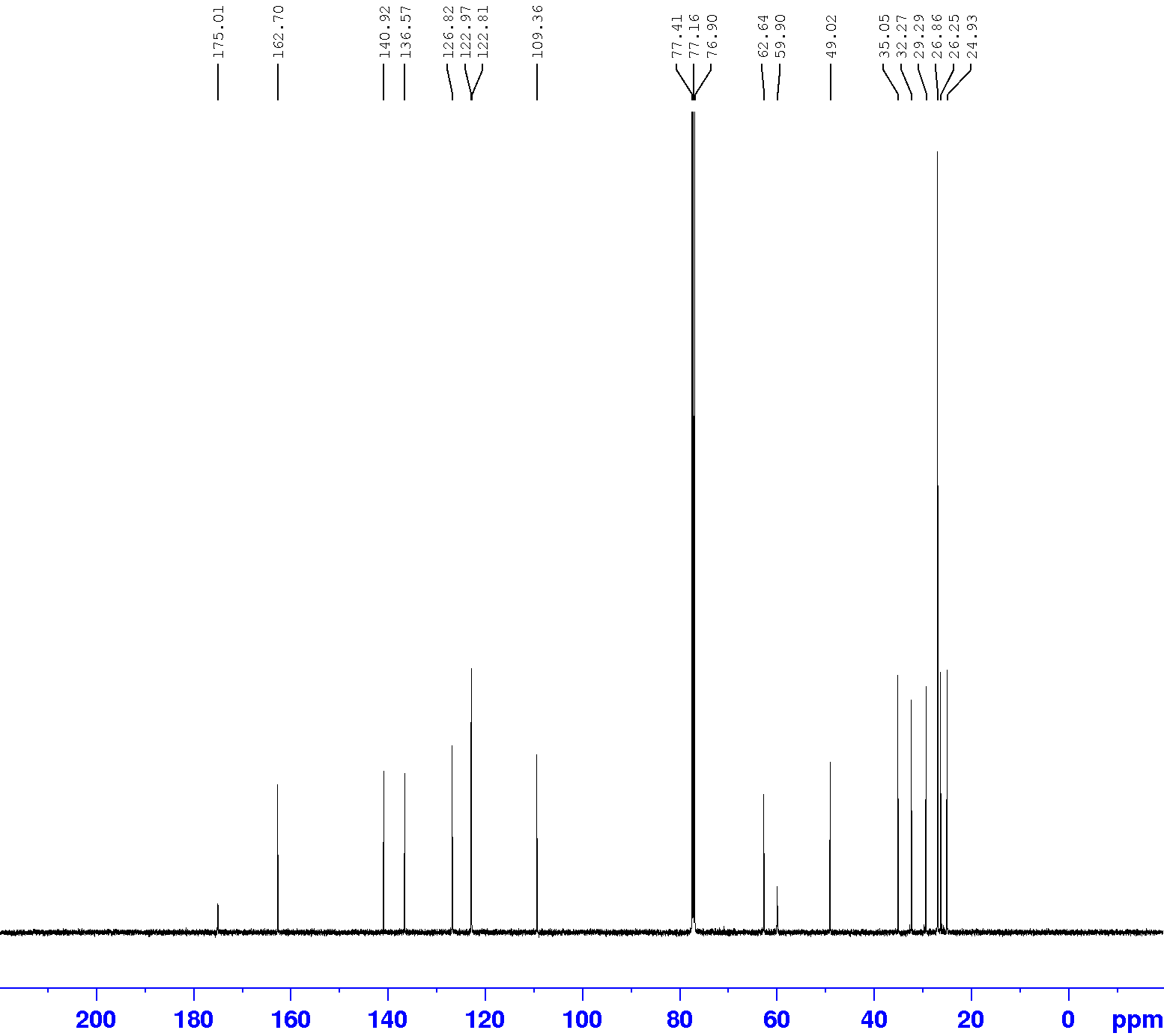

**5OH-ADB-HEXINACA**

^1^H-NMR (CDCl_3_, 500 MHz) δ: 8.28 (d, *J* = 8.2 Hz, 1H), 7.73 (t, *J* = 10.4 Hz, 1H), 7.38-7.43 (m, 2H), 7.24-7.27 (m, 1H), 6.40 (bs, 1H), 5.70 (d, *J* = 8.9 Hz, 1H), 4.61 (d, *J* = 9.4 Hz, 1H), 4.41 (quart, *J* = 6.6 Hz, 2H), 3.73-3.83 (m, 1H), 1.90-2.03 (m, 2H), 1.75 (bs, 1H), 1.37-1.59 (m, 4H), 1.16-1.18 (m, 3H), 1.14 (s, 9H). ^13^C-NMR (CDCl_3_, 126 MHz) δ: 173.1, 162.8, 140.9, 136.6, 126.8, 123.0, 122.8, 122.7, 109.4, 67.9, 59.8, 49.3, 38.5, 34.8, 29.5, 26.9, 23.7, 23.0.


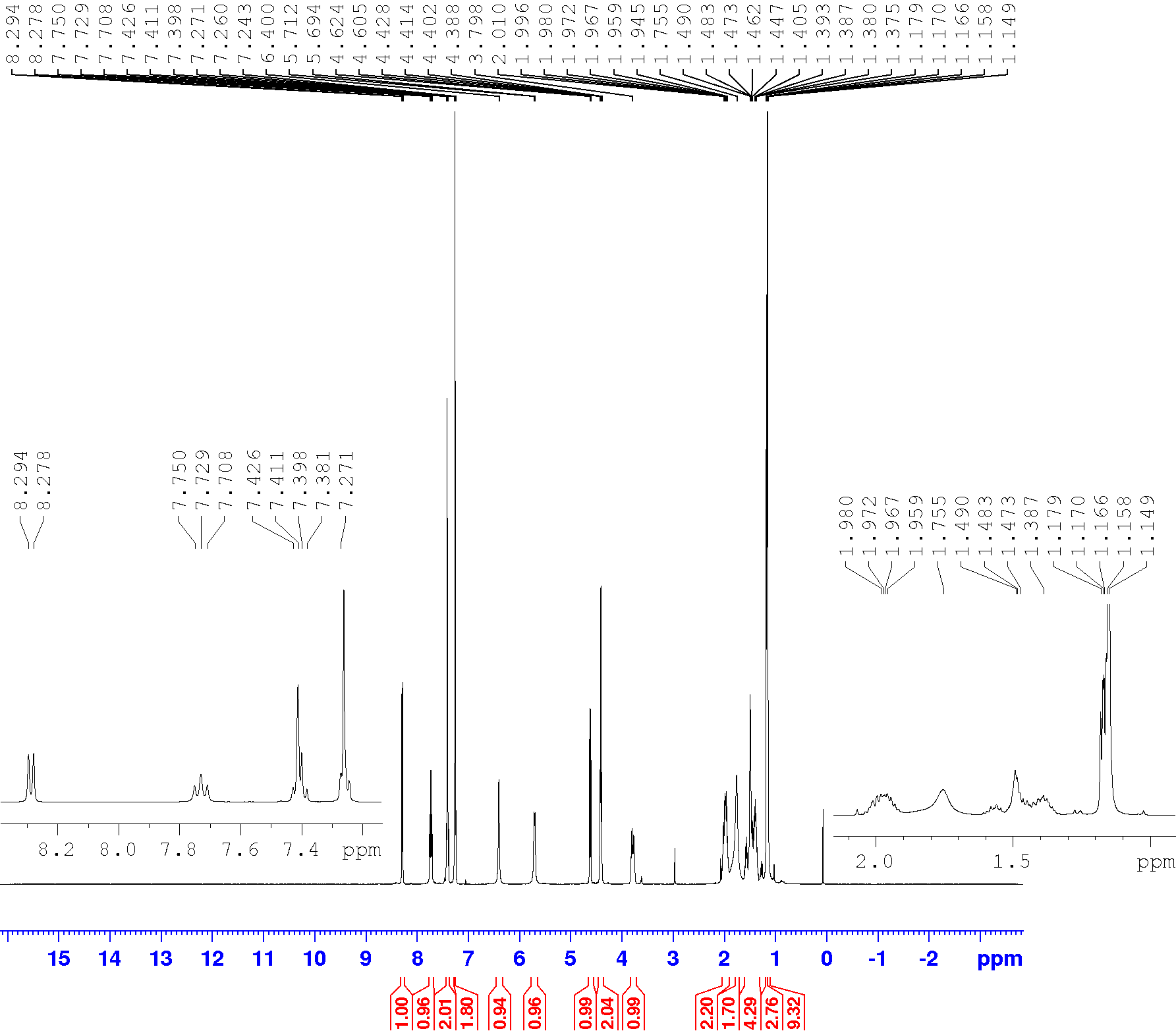


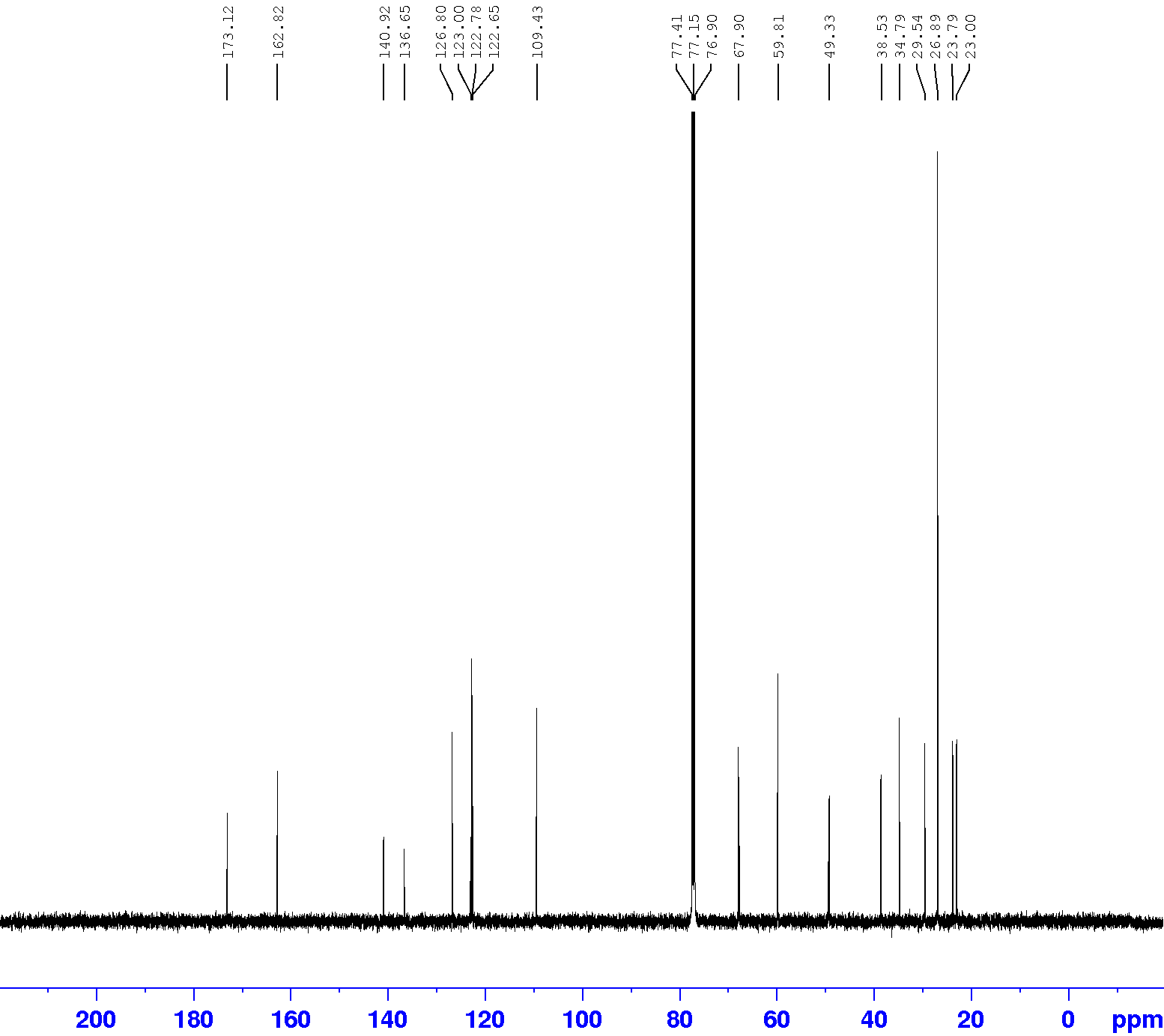

**5OH-COOH-ADB-HEXINACA**

^1^H-NMR (CD_3_OD, 500 MHz) δ: 8.20 (d, *J* = 8.2 Hz, 1H), 7.64 (d, *J* = 8.5 Hz, 1H), 7.45 (t, *J* = 7.7 Hz, 1H), 7.28 (t, *J* = 7.6, 1H), 4.56 (s, 1H), 4.51 (t, *J* = 7.0 Hz, 2H), 3.66-3.68 (m, 1H), 1.94-2.00 (m, 2H), 1.28-1.53 (m, 4H), 1.11 (s, 9H), 1.10 (s, 3H). ^13^C-NMR (CD_3_OD, 126 MHz) δ: 174.1, 164.2, 142.4, 137.4, 128.0, 123.9, 123.8, 123.0, 111.1, 68.3, 61.1, 50.3, 39.5, 35.6, 30.8, 27.2, 24.0, 23.5.


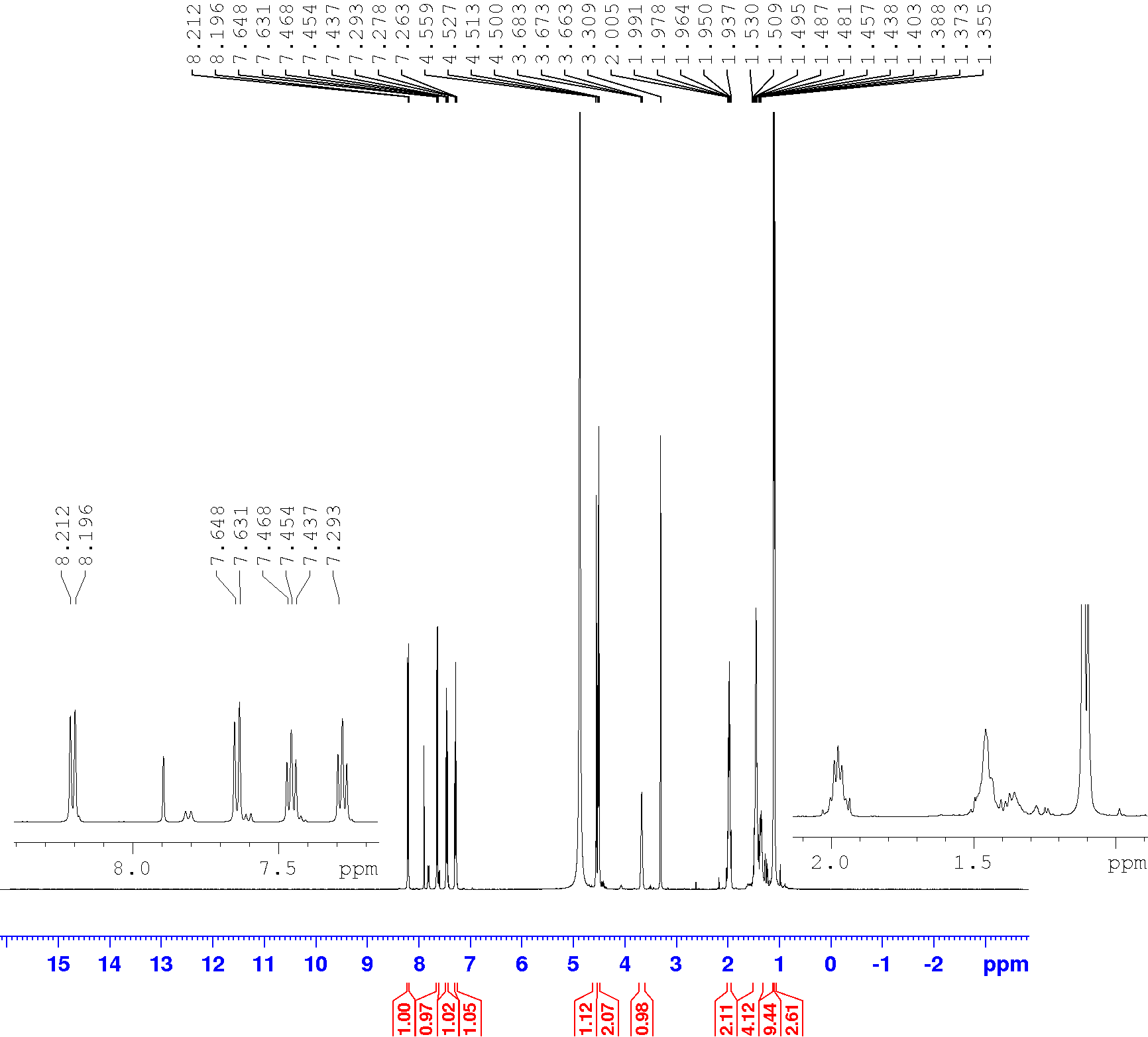


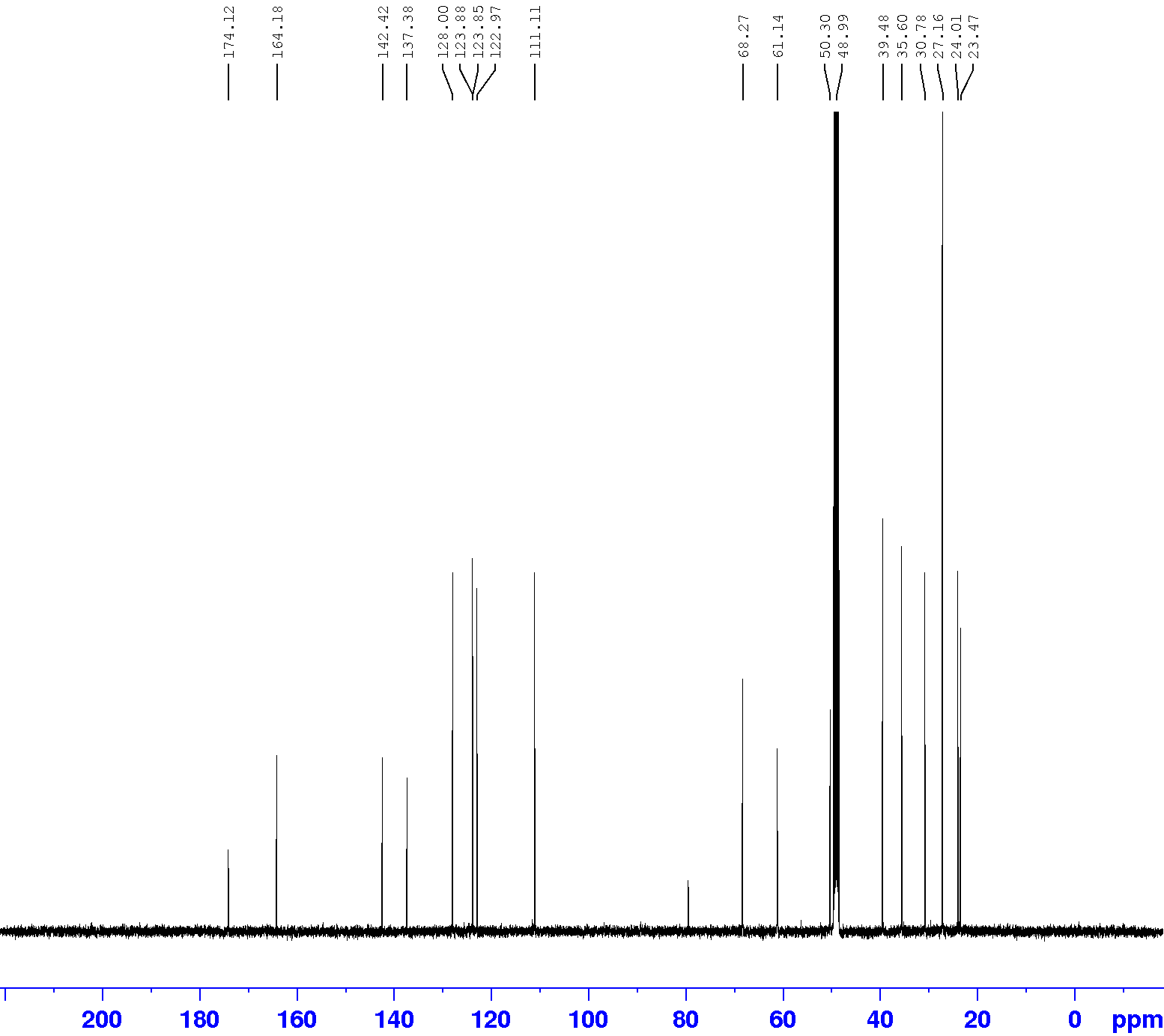

**4OH-ADB-HEXINACA**

^1^H-NMR (CD_3_OD, 500 MHz) δ: 8.28 (d, *J* = 8.2 Hz, 1H), 7.69 (d, *J* = 9.4 Hz, 1H), 7.38-7.44 (m, 2H), 7.24-7.27 (m, 1H), 6.37 (bs, 1H), 5.70 (bs, 1H), 4.61 (d, *J* = 9.5 Hz, 1H), 4.43 (t, *J* = 7.2 Hz, 2H), 3.54-3.60 (m, 1H), 1.98-2.17 (m, 2H), 1.75 (bs, 1H), 1.40-1.54 (m, 4H), 1.28-1.53 (m, 4H), 1.15 (s, 9H), 0.91 (dt, *J* = 7.5, 3.1 Hz 3H). ^13^C-NMR (CD_3_OD, 126 MHz) δ: 173.0, 162.8, 141.0, 136.7, 126.8, 123.0, 123.8, 122.6, 109.5, 72.8, 59.8, 49.5, 34.8, 33.9, 30.5, 26.9, 26.1, 10.0.


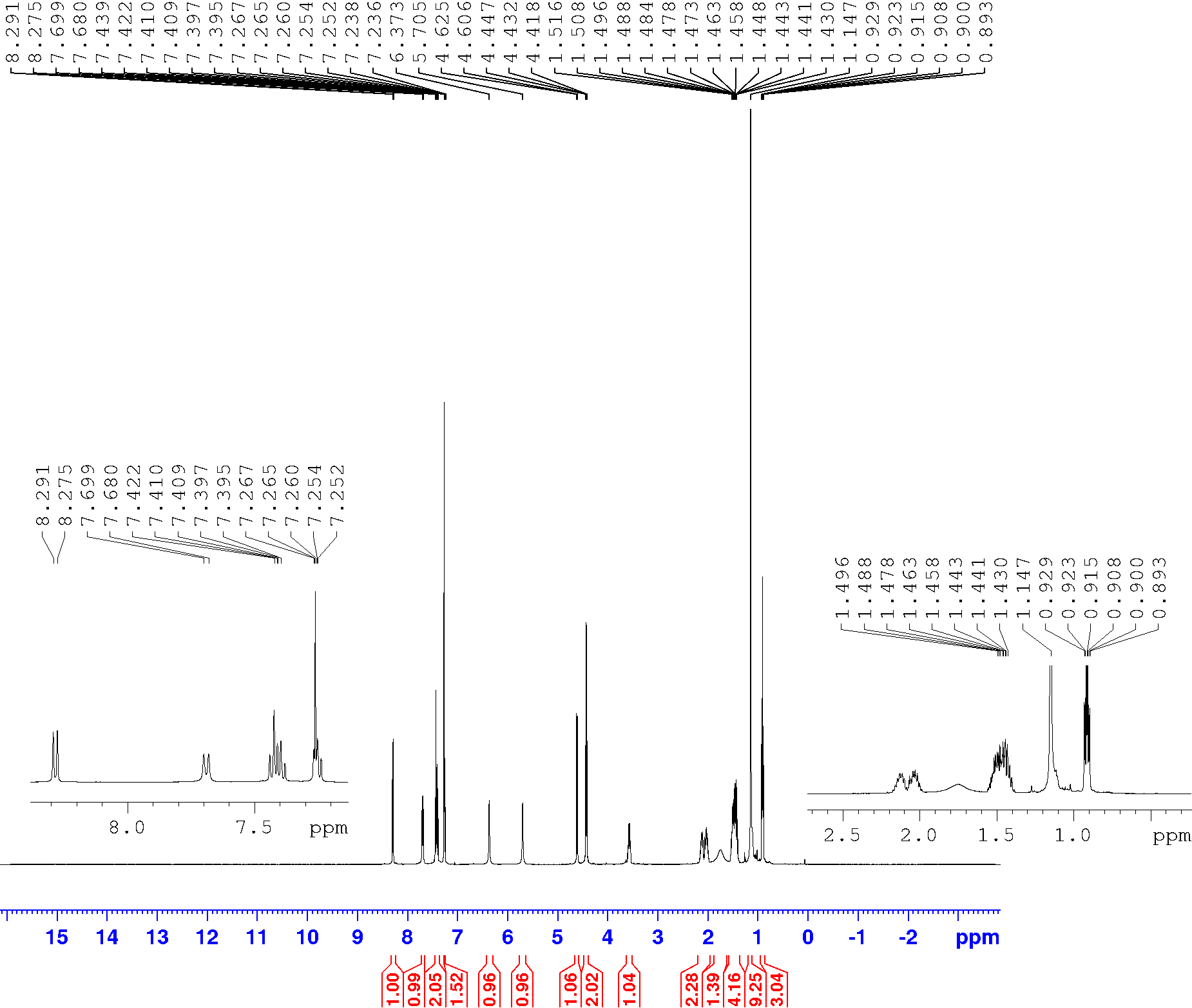


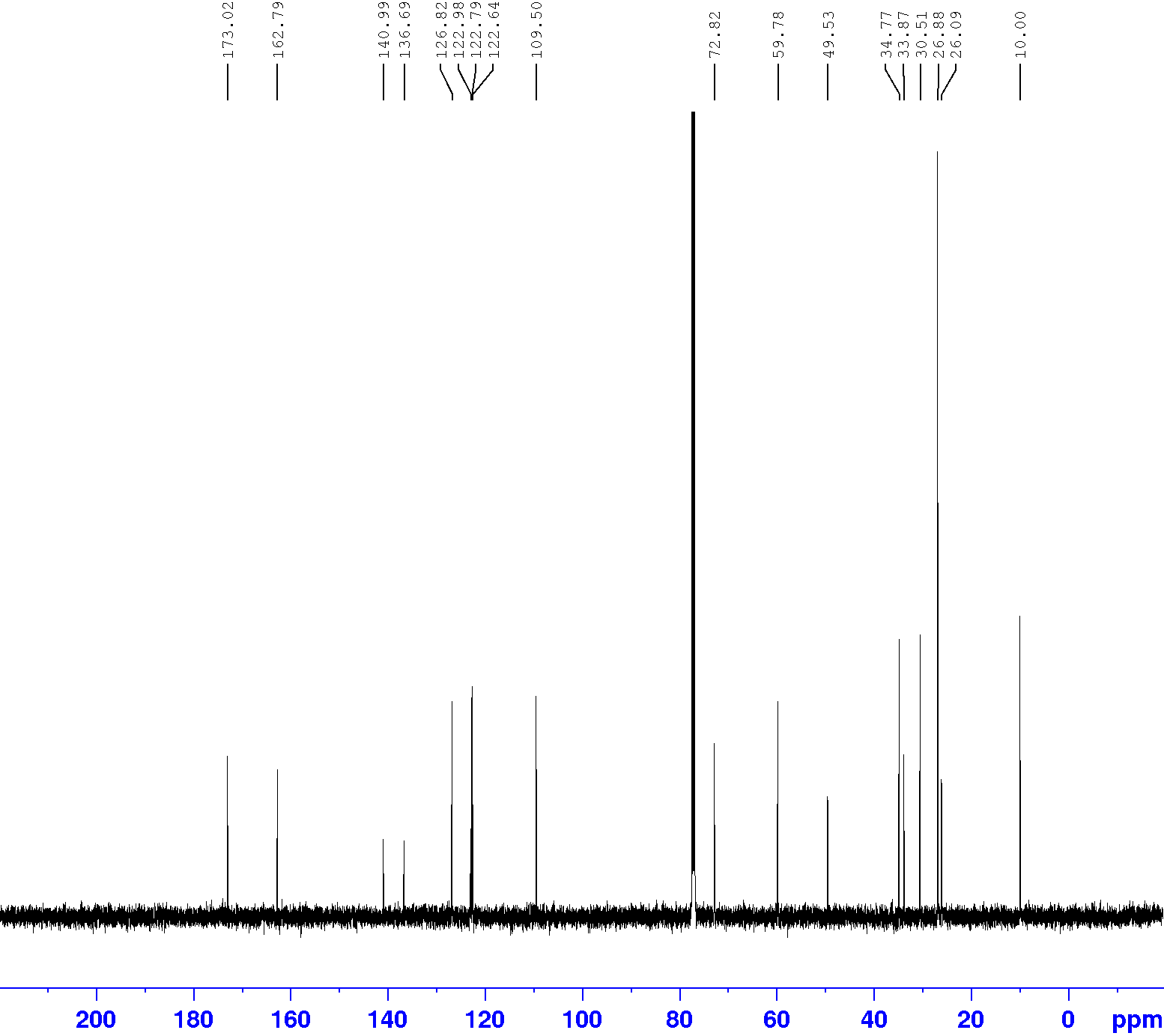

**5OXO-ADB-HEXINACA**

^1^H-NMR (CD_3_OD, 500 MHz) δ: 8.21 (d, *J* = 8.2 Hz, 1H), 7.65 (d, *J* = 8.5 Hz, 1H), 7.46 (t, *J* = 7.7 Hz, 1H), 7.28 (t, *J* = 7.7 Hz, 1H), 4.53 (s, 1H), 4.51 (t, *J* = 7.0 Hz, 2H), 2.53 (t, *J* = 7.2 Hz, 2H), 2.09 (s, 3H), 1.96 (quin, J = 7.3 Hz, 2H), 1.57 (quin, J = 7.5 Hz, 2H) 1.10 (s, 9H). ^13^C-NMR (CD_3_OD, 126 MHz) δ: 211.4, 175.1, 164.1, 142.5, 137.6, 128.0, 123.9, 123.8, 123.0, 111.1, 61.0, 50.1, 43.4, 35.7, 30.1, 29.8, 27.2, 21.9.


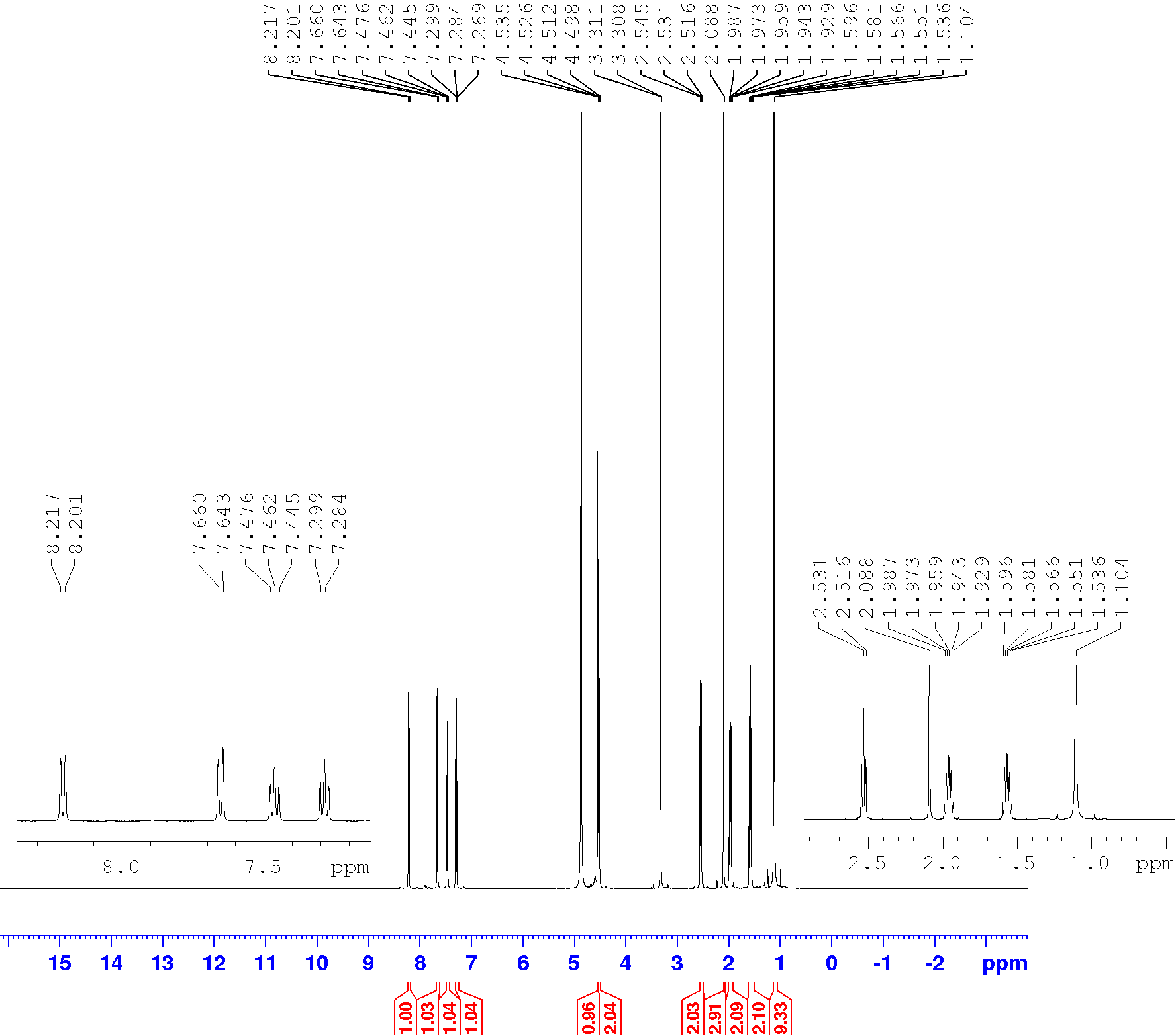


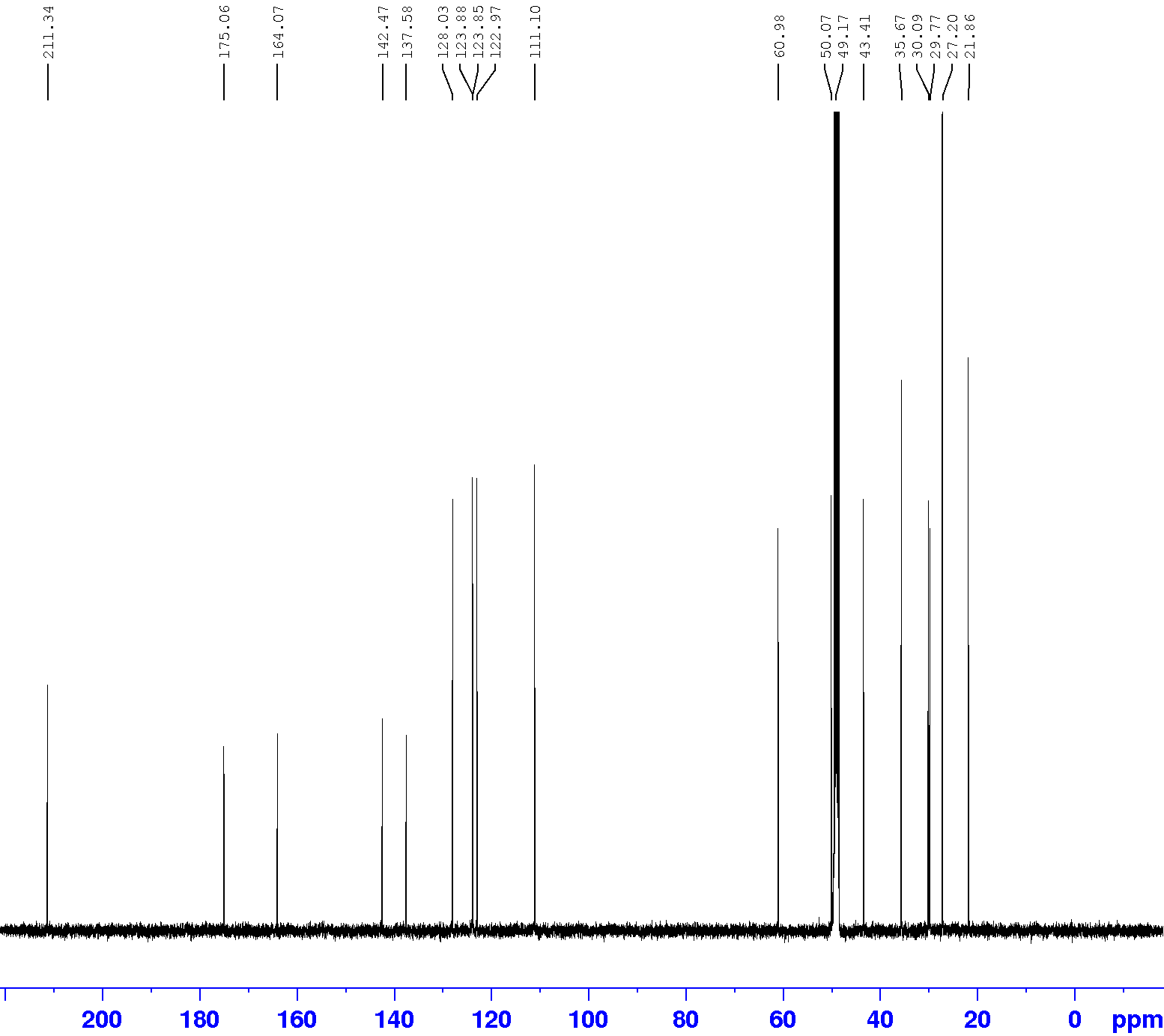

**5OXO-COOH-ADB-HEXINACA**

^1^H-NMR (CD_3_OD, 500 MHz) δ: 8.22 (d, *J* = 8.2 Hz, 1H), 7.67 (d, *J* = 8.6 Hz, 1H), 7.48 (t, *J* = 7.7 Hz, 1H), 7.30 (t, *J* = 7.3 Hz, 1H), 4.57 (s, 1H), 4.54 (t, *J* = 7.0 Hz, 2H), 2.55 (t, *J* = 7.3 Hz, 2H), 2.10 (s, 3H), 1.99 (quin, J = 7.4 Hz, 2H), 1.59 (quin, J = 7.5 Hz, 2H), 1.14 (s, 9H). ^13^C-NMR (CD_3_OD, 126 MHz) δ: 211.4, 174.3, 164.2, 142.4, 137.5, 128.0, 123.9, 123.8, 123.0, 111.1, 61.3, 50.1, 43.4, 35.6, 30.1, 29.8, 27.2, 21.9.


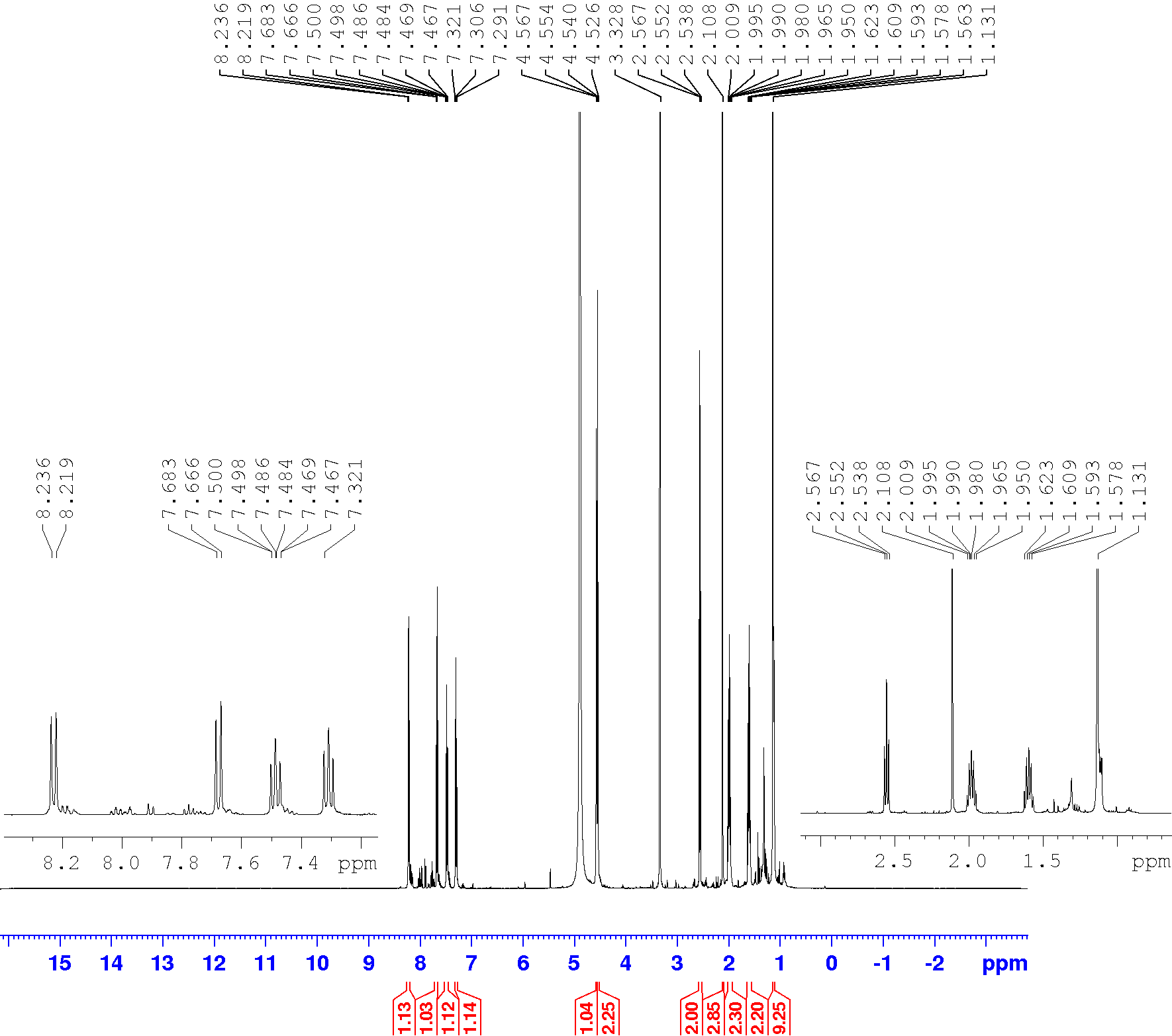


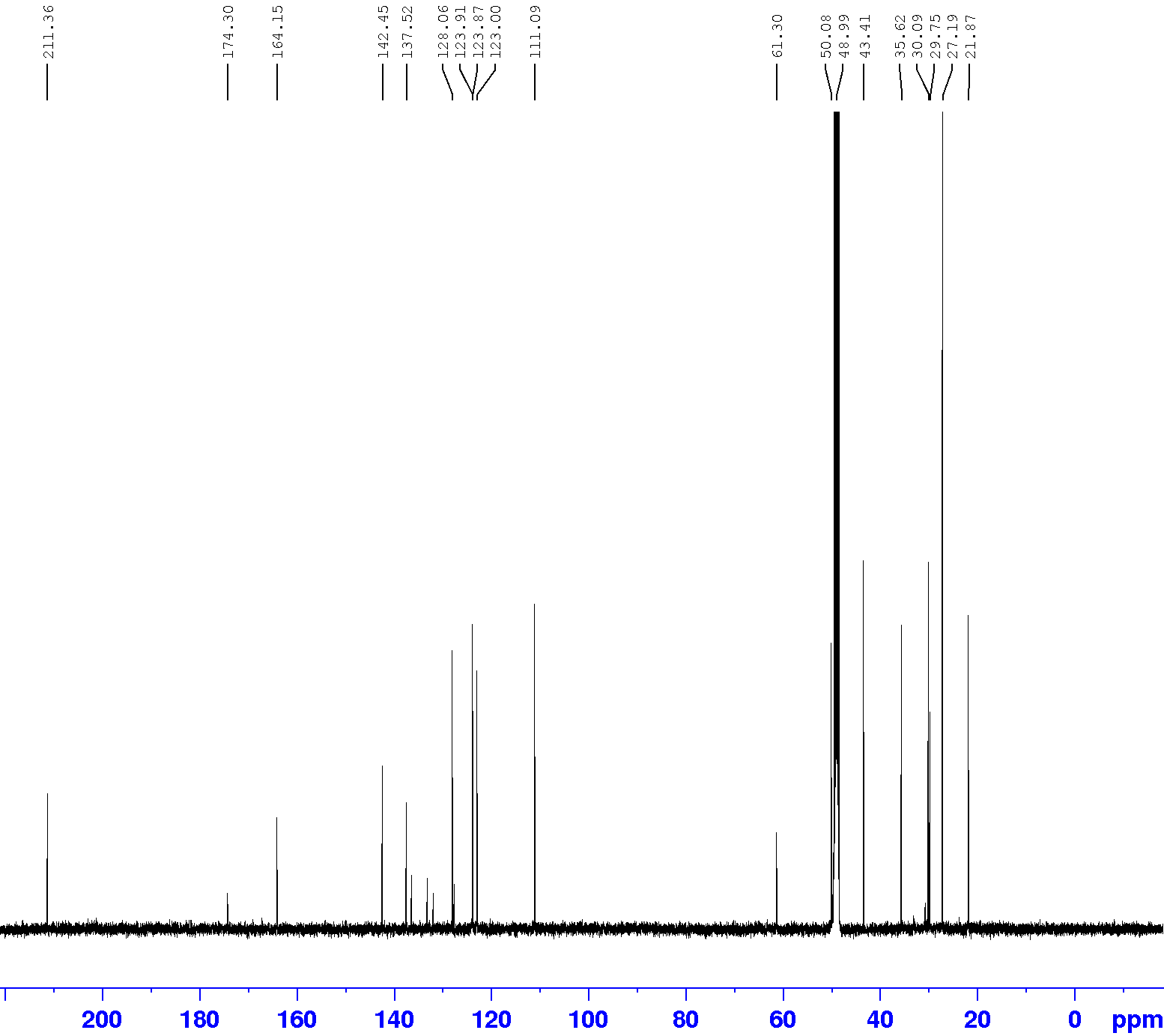

**4OXO-ADB-HEXINACA**

^1^H-NMR (CD_3_OD, 500 MHz) δ: 8.30 (d, *J* = 8.2 Hz, 1H), 7.65 (d, *J* = 9.4 Hz, 1H), 7.40-7.46 (m, 2H), 7.27-7.28 (m, 1H), 6.08 (bs, 1H), 5.55 (bs, 1H), 4.56 (d, *J* = 9.5 Hz, 1H), 4.45 (td, *J* = 7.2 Hz, 2H), 2.35-2.43 (m, 4H), 2.22 (q, *J*  = Hz, 2H), 1.15 (s, 9H), 1.03 (t, *J* = 7.5, 3.1 Hz 3H). ^13^C-NMR (CD_3_OD, 126 MHz) δ: 210.6, 172.8, 162.7, 141.1, 136.9, 127.0, 123.0, 122.9, 122.7, 109.5, 59.8, 48.4, 39.5, 36.2, 34.8, 26.9, 23.7, 7.9.


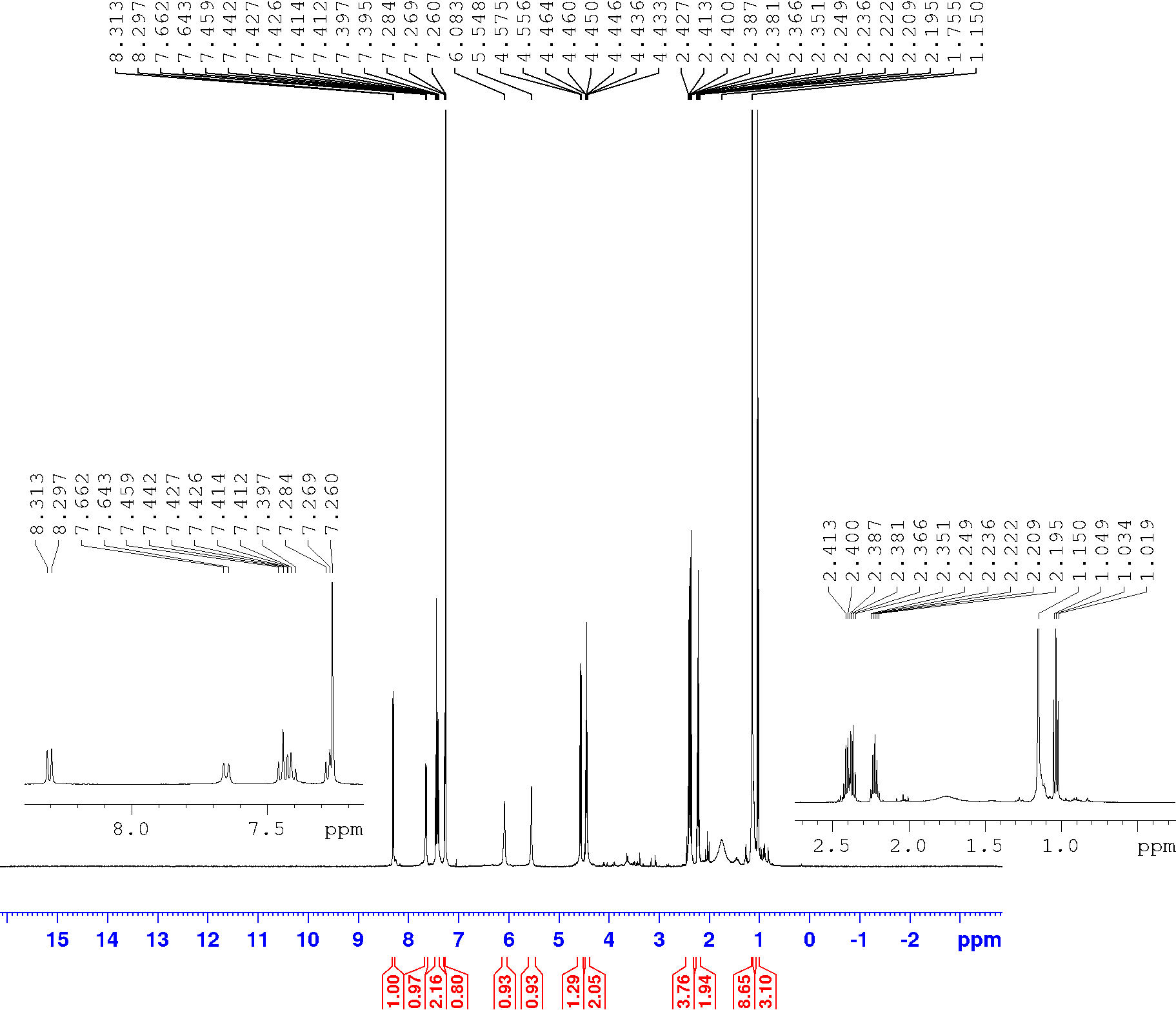


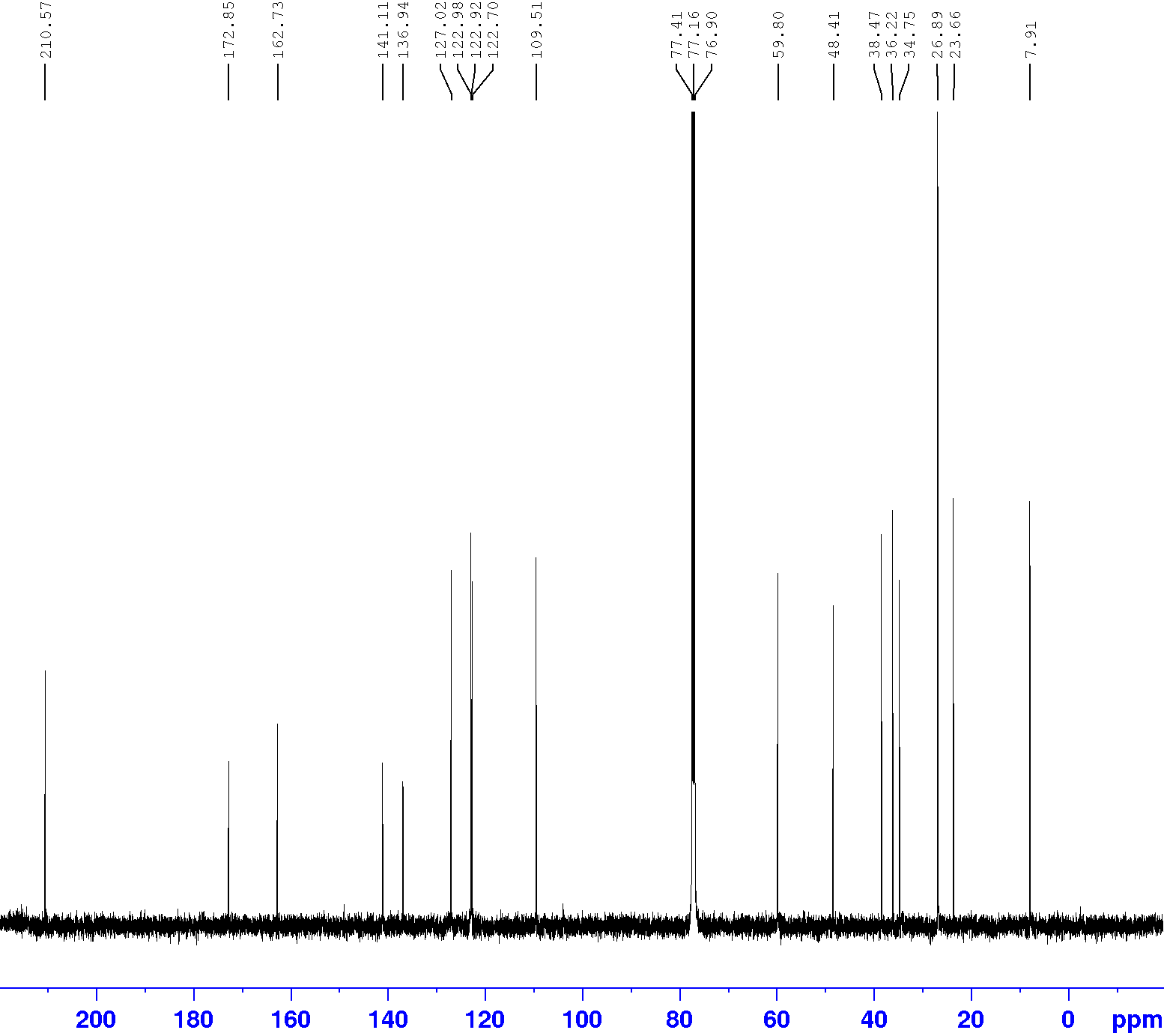

**6OXO-ADB-HEXINACA**

^1^H-NMR (CDCl_3_, 500 MHz) δ: 9.75 (s, 1H), 8.28 (d, *J* = 8.2 Hz, 1H), 7.69 (d, *J* = 9.4 Hz, 1H), 7.40-7.41 (m, 2H), 7.24-7.28 (m, 1H), 6.40 (bs, 1H), 5.58 (bs, 1H), 4.63 (d, *J* = 9.5 Hz, 1H), 4.40 (t, *J* = 7.1 Hz, 2H), 2.44 (t, *J* = 7.2 Hz, 2H), 1.98 (quin, J = 7.4 Hz, 2H), 1.68 (quin, J = 7.5 Hz, 2H), 1.37 (quin, J = 7.7 Hz, 2H), 1.15 (s, 9H). ^13^C-NMR (CDCl_3_, 126 MHz) δ: 202.4, 173.0, 162.8, 141.0, 136.8, 126.9, 123.0, 122.8, 122.7, 109.4, 59.8, 49.2, 43.8, 34.8, 29.6, 26.9, 26.4, 21.6.


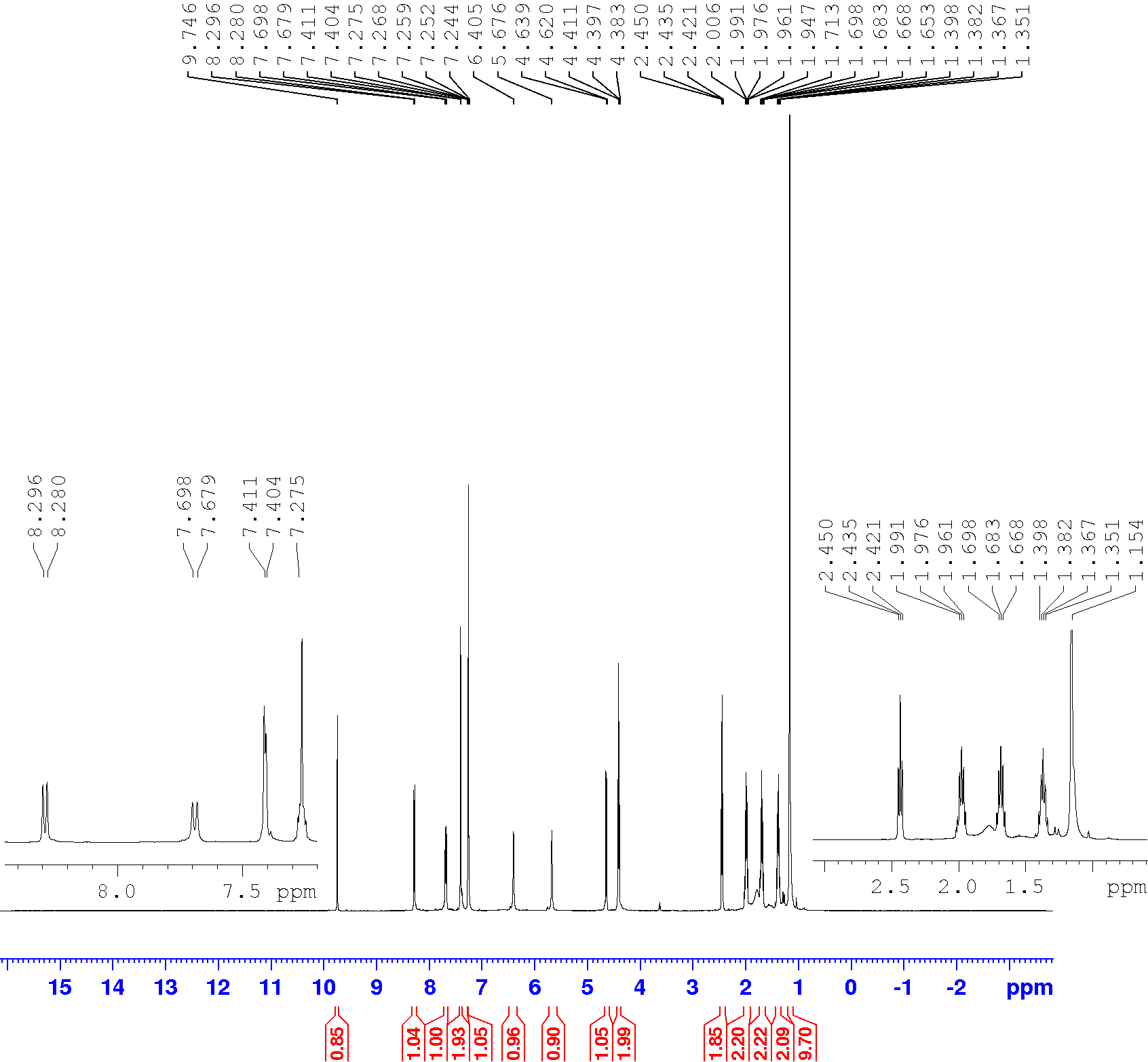


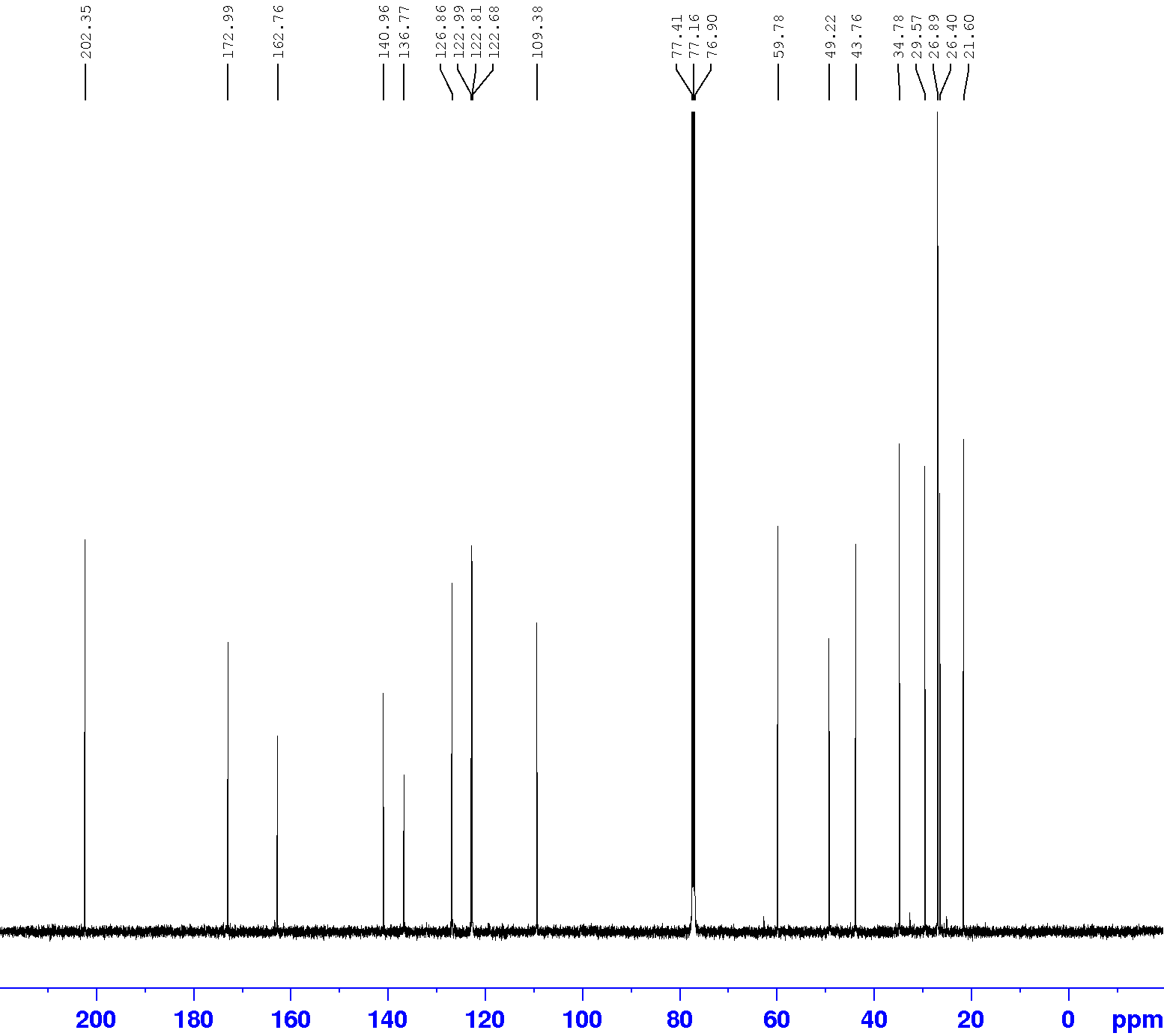

COOH

5OXO-COOH

5OXO

5OH

6OH

4OH

4OXO

M6

M7

M8

M9

M11

M16

M13
