# Supplementary material for: The metabolic profile of the synthetic cannabinoid receptor agonist ADB-HEXINACA using human hepatocytes, LC–QTOF-MS and synthesized reference standards
Source: J Anal Toxicol. 2023 Sep 25;47(9):826–34. doi: 10.1093/jat/bkad065 (PMC10714907; doi:10.1093/jat/bkad065)
Supplement: bkad065_Supp [file bkad065_supp.zip › jat-23-3987-File007.pptx]

## Slide 1
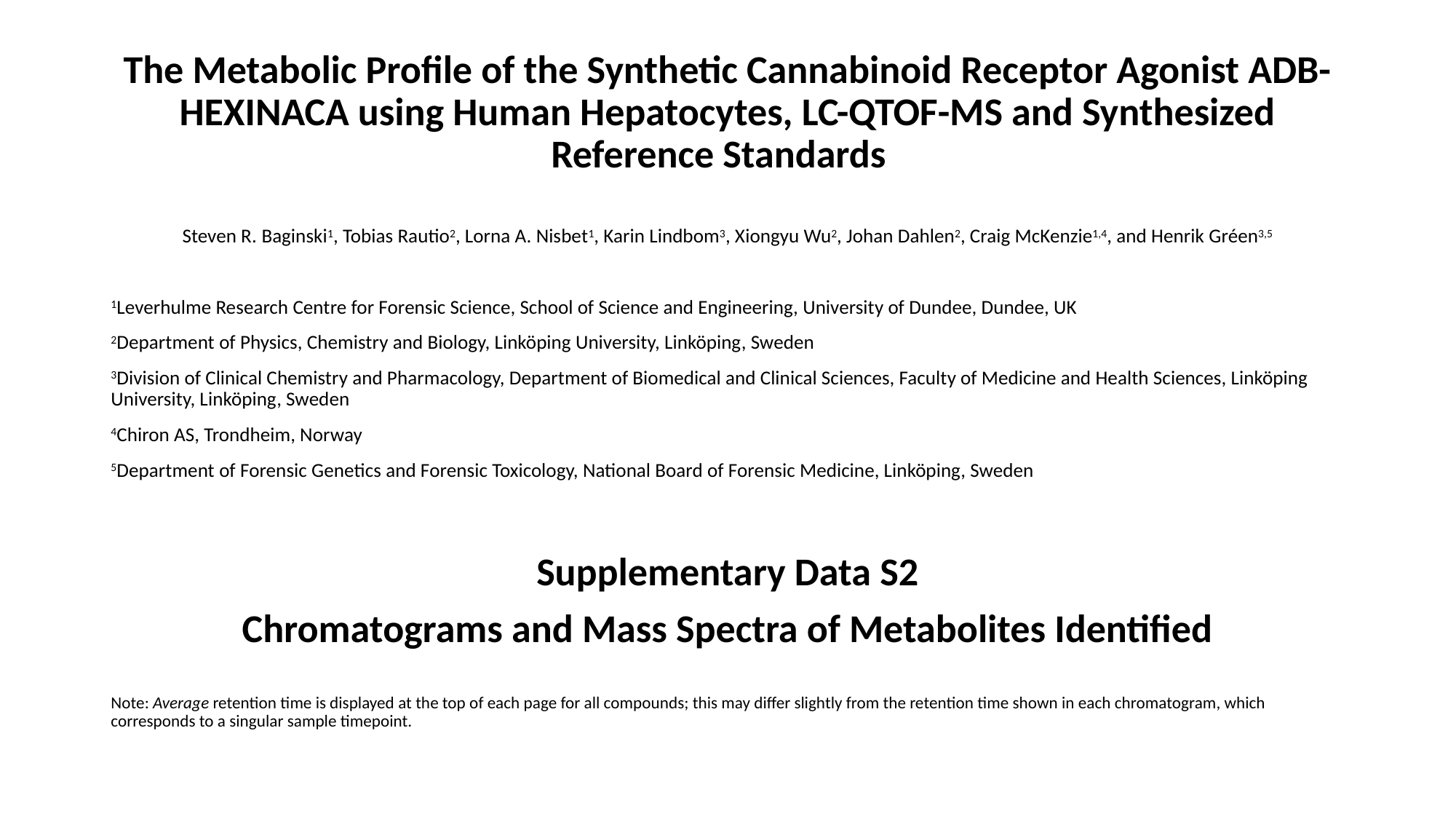

The Metabolic Profile of the Synthetic Cannabinoid Receptor Agonist ADB-HEXINACA using Human Hepatocytes, LC-QTOF-MS and Synthesized Reference Standards
Steven R. Baginski1, Tobias Rautio2, Lorna A. Nisbet1, Karin Lindbom3, Xiongyu Wu2, Johan Dahlen2, Craig McKenzie1,4, and Henrik Gréen3,5
1Leverhulme Research Centre for Forensic Science, School of Science and Engineering, University of Dundee, Dundee, UK
2Department of Physics, Chemistry and Biology, Linköping University, Linköping, Sweden
3Division of Clinical Chemistry and Pharmacology, Department of Biomedical and Clinical Sciences, Faculty of Medicine and Health Sciences, Linköping University, Linköping, Sweden
4Chiron AS, Trondheim, Norway
5Department of Forensic Genetics and Forensic Toxicology, National Board of Forensic Medicine, Linköping, Sweden
Supplementary Data S2
Chromatograms and Mass Spectra of Metabolites Identified
Note: Average retention time is displayed at the top of each page for all compounds; this may differ slightly from the retention time shown in each chromatogram, which corresponds to a singular sample timepoint.

## Slide 2
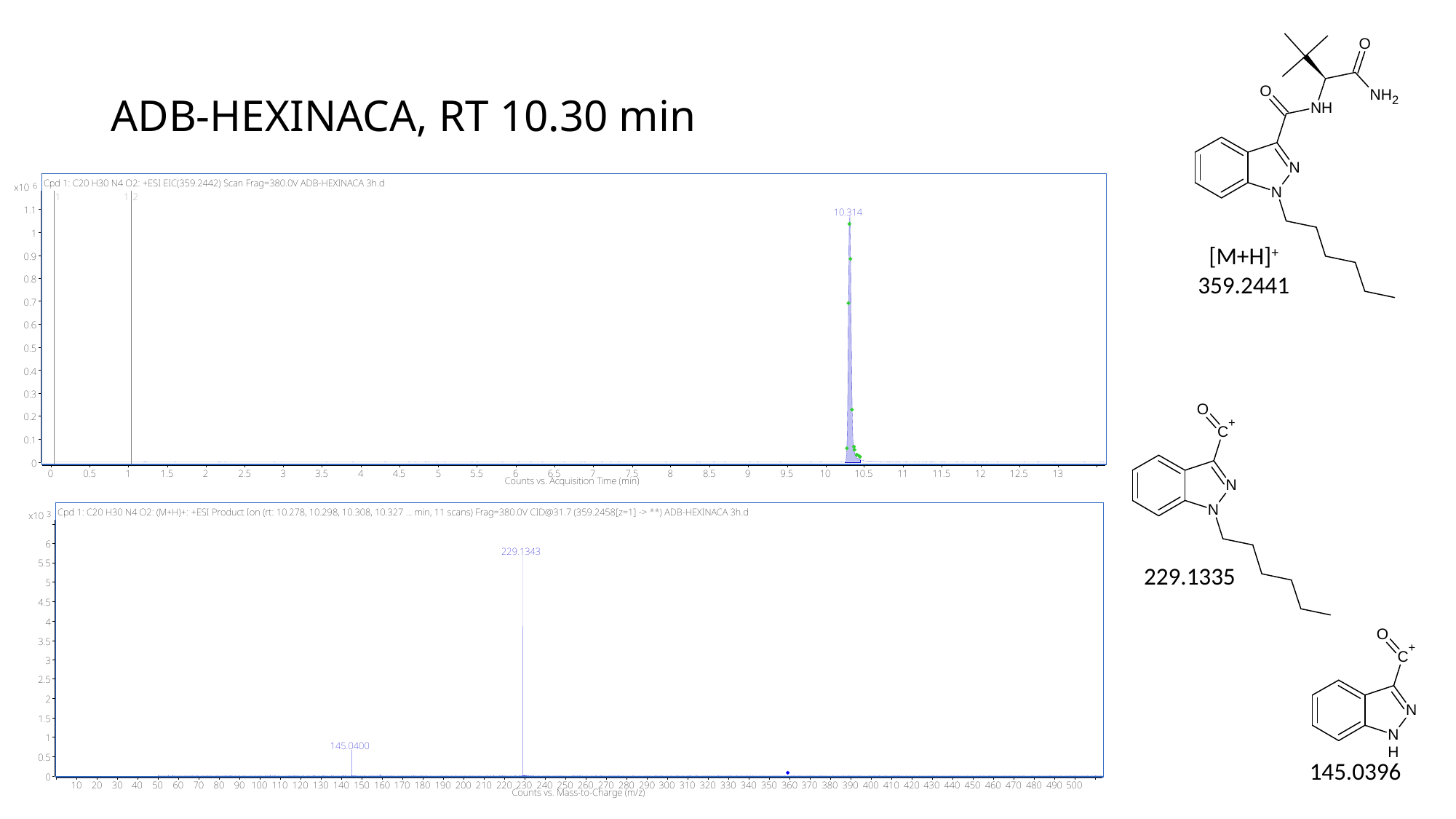

[M+H]+
359.2441
145.0396
# ADB-HEXINACA, RT 10.30 min
229.1335

## Slide 3
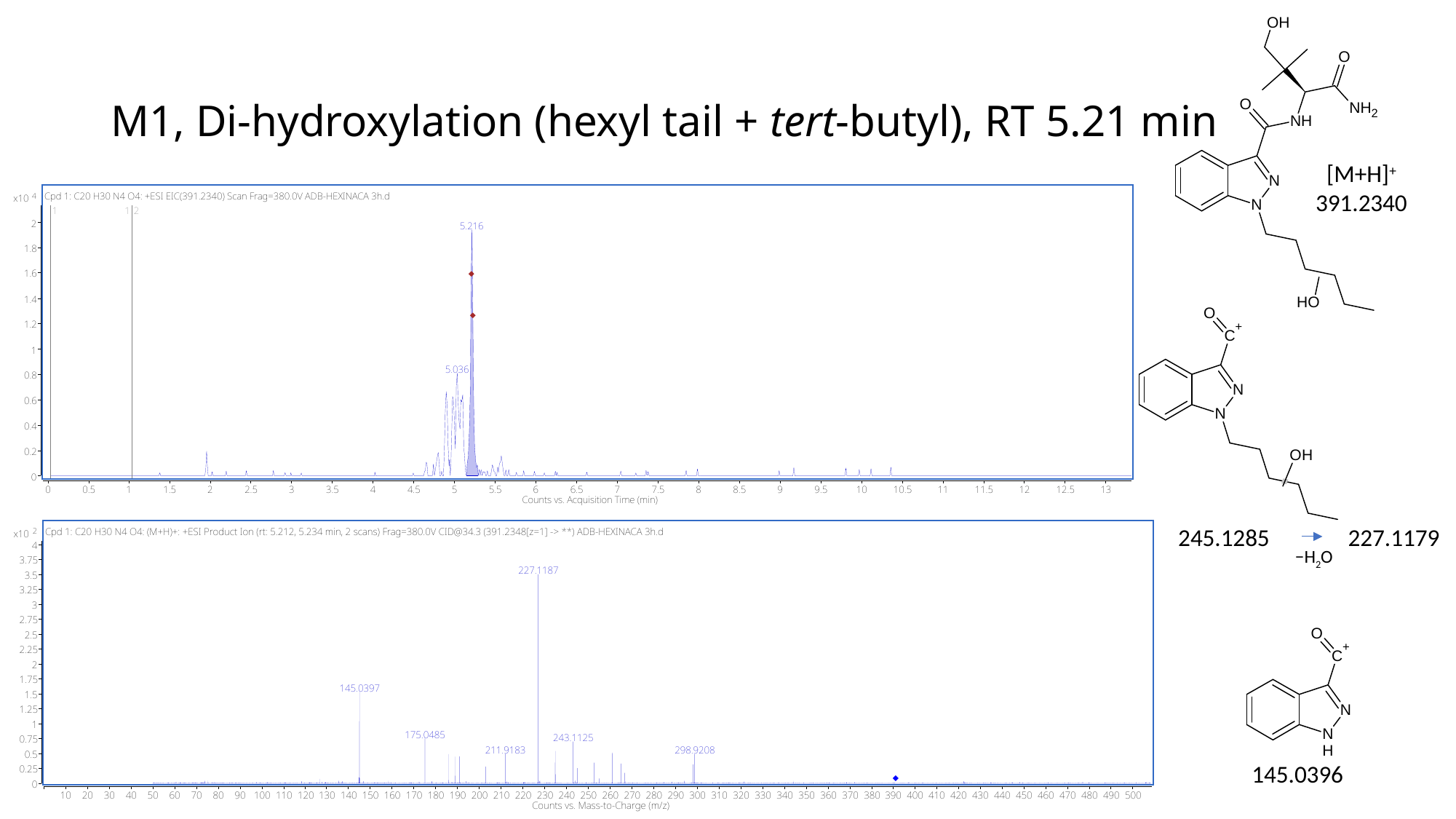

[M+H]+
391.2340
245.1285
227.1179
−H2O
145.0396
# M1, Di-hydroxylation (hexyl tail + tert-butyl), RT 5.21 min

## Slide 4
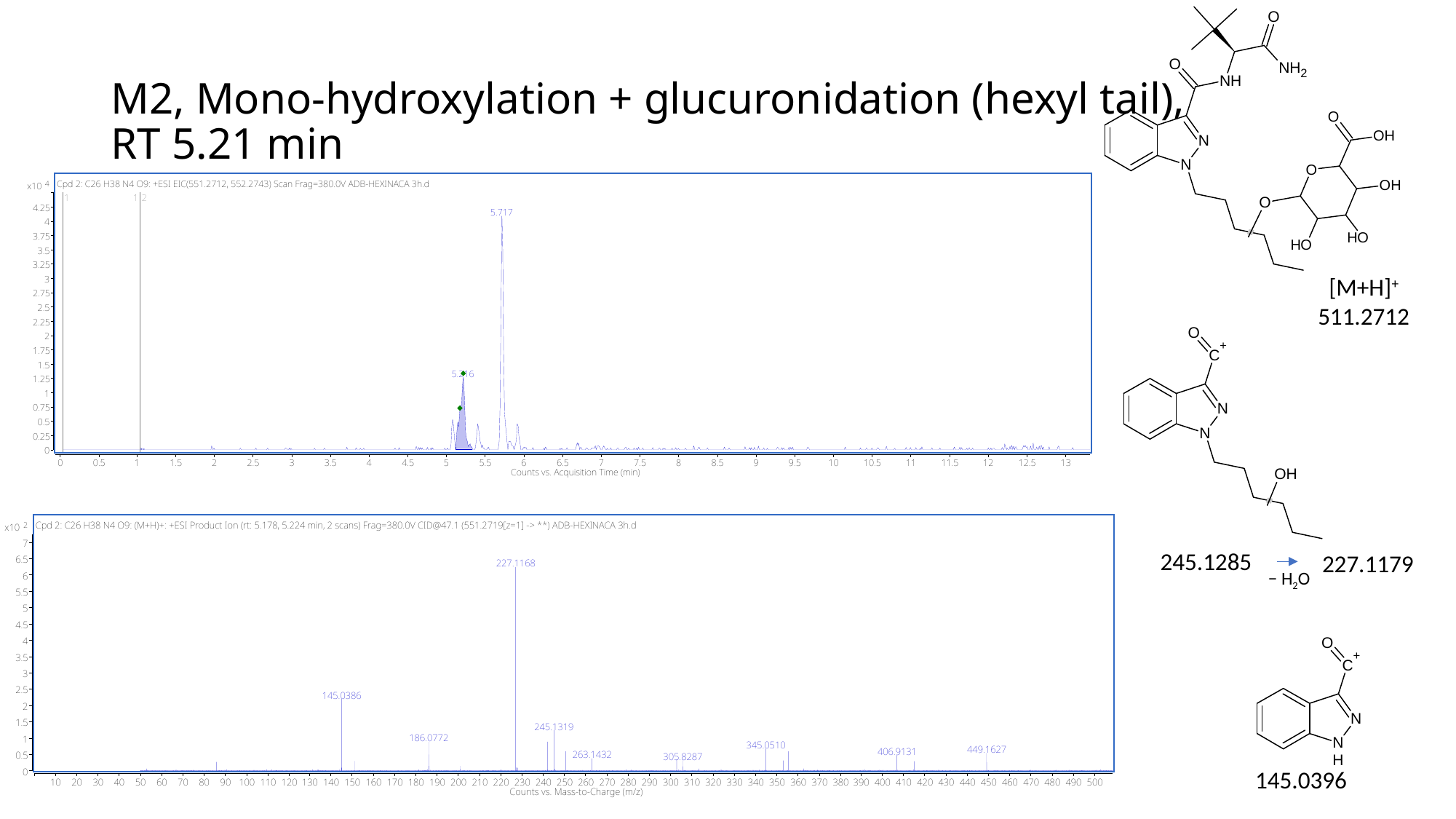

[M+H]+
511.2712
245.1285
227.1179
− H2O
145.0396
# M2, Mono-hydroxylation + glucuronidation (hexyl tail), RT 5.21 min

## Slide 5
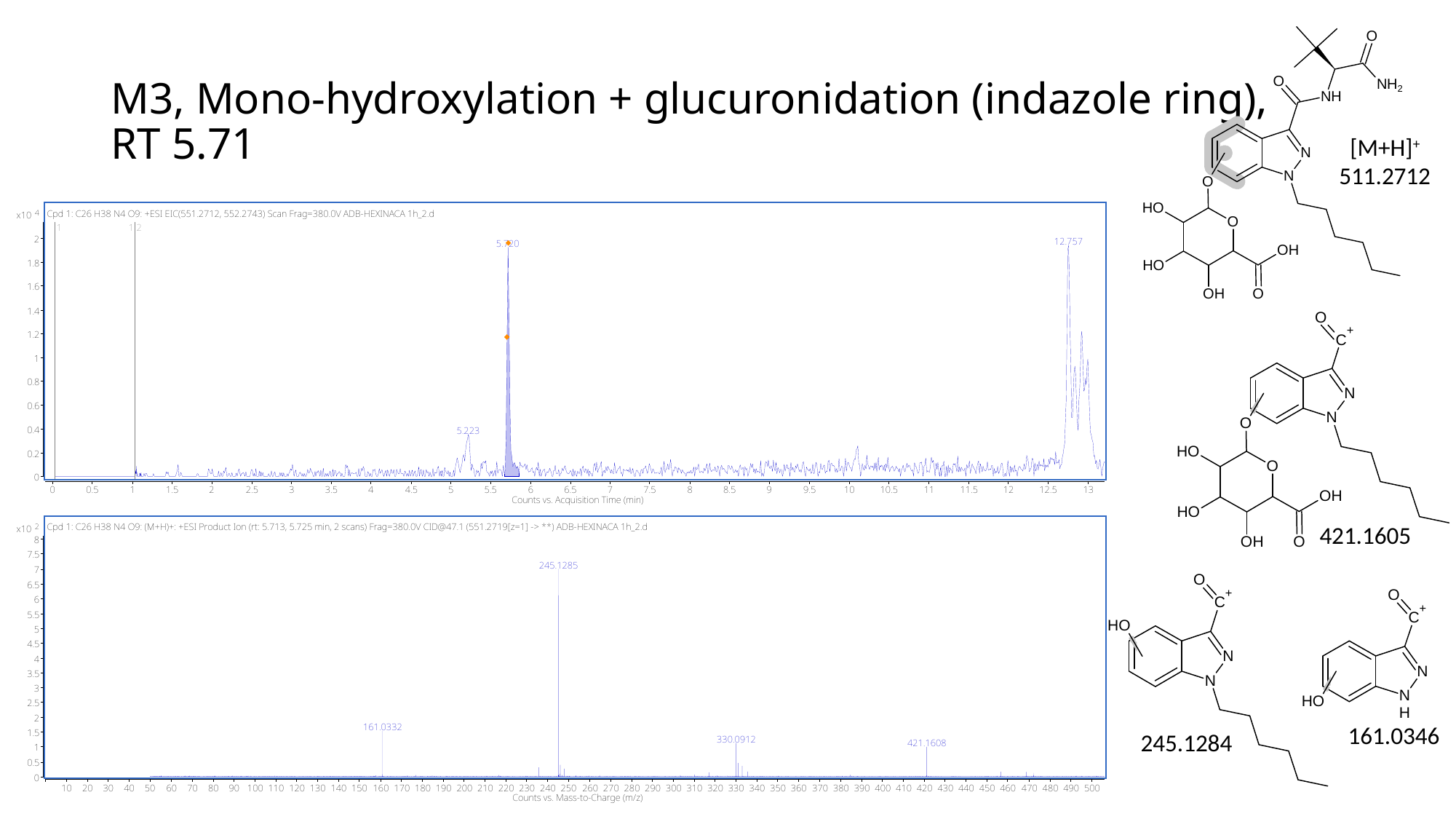

[M+H]+
511.2712
421.1605
161.0346
245.1284
# M3, Mono-hydroxylation + glucuronidation (indazole ring), RT 5.71

## Slide 6
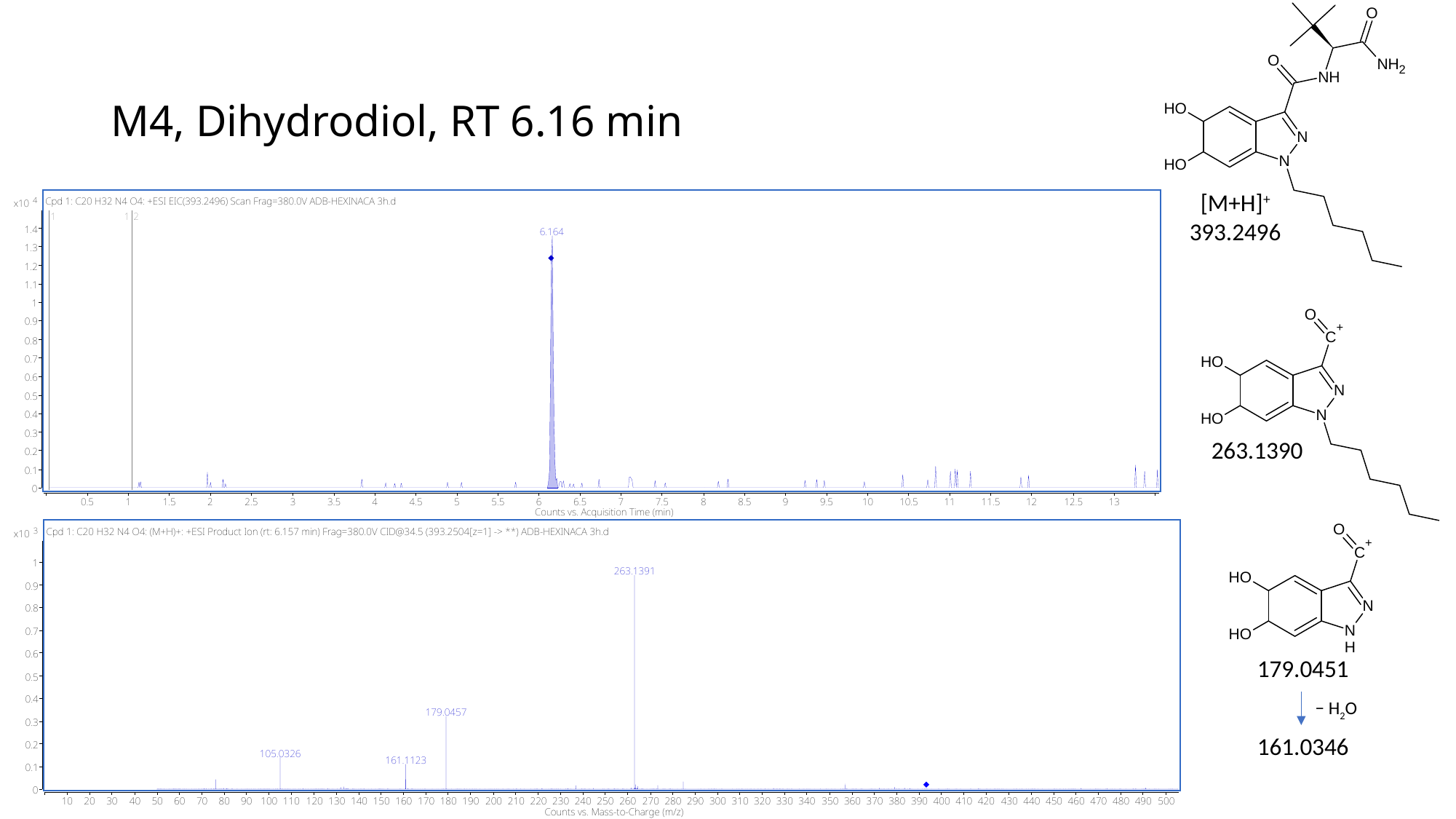

[M+H]+
393.2496
263.1390
179.0451
− H2O
161.0346
# M4, Dihydrodiol, RT 6.16 min

## Slide 7
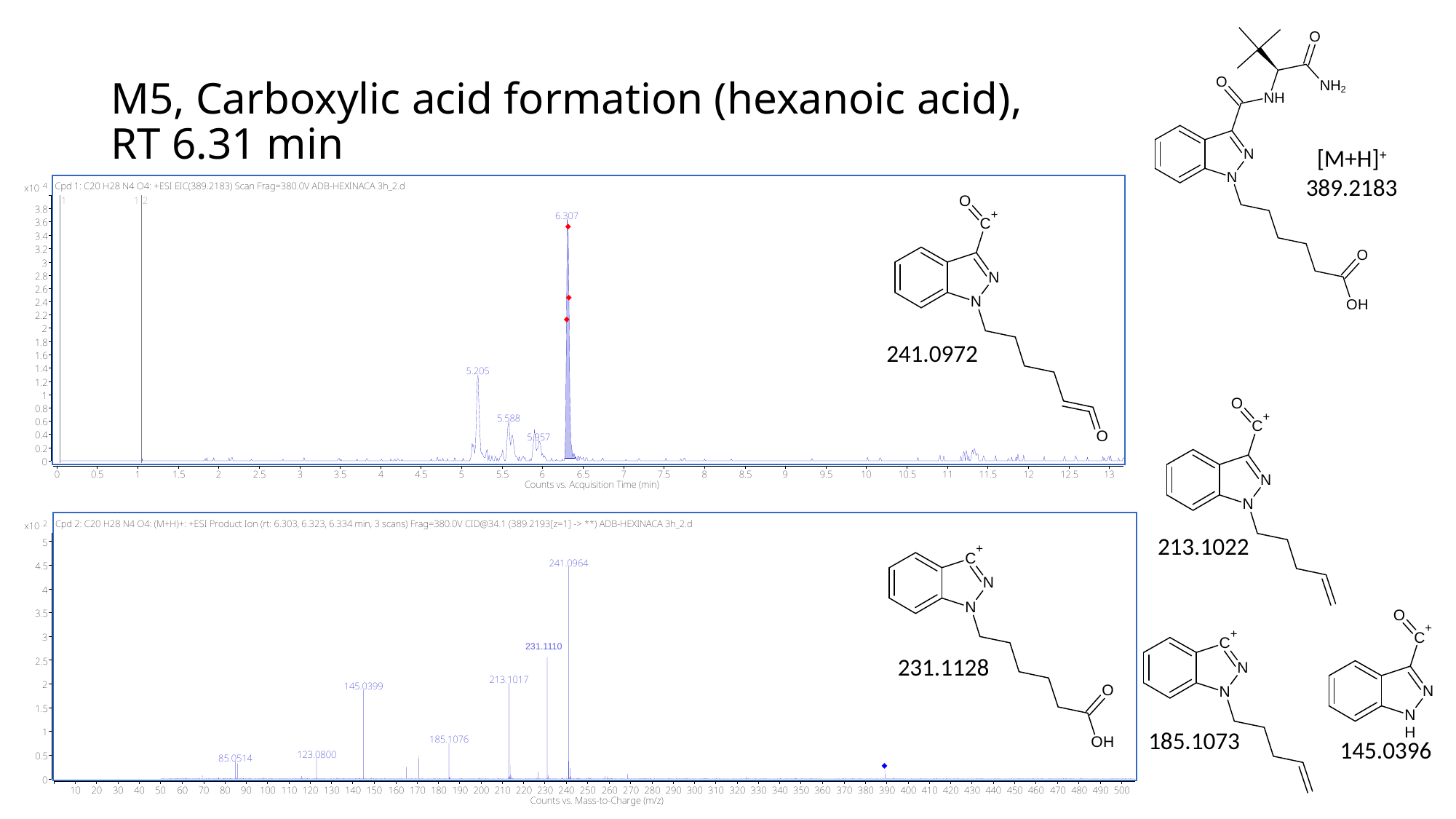

[M+H]+
389.2183
241.0972
213.1022
231.1128
185.1073
145.0396
# M5, Carboxylic acid formation (hexanoic acid), RT 6.31 min
231.1110

## Slide 8
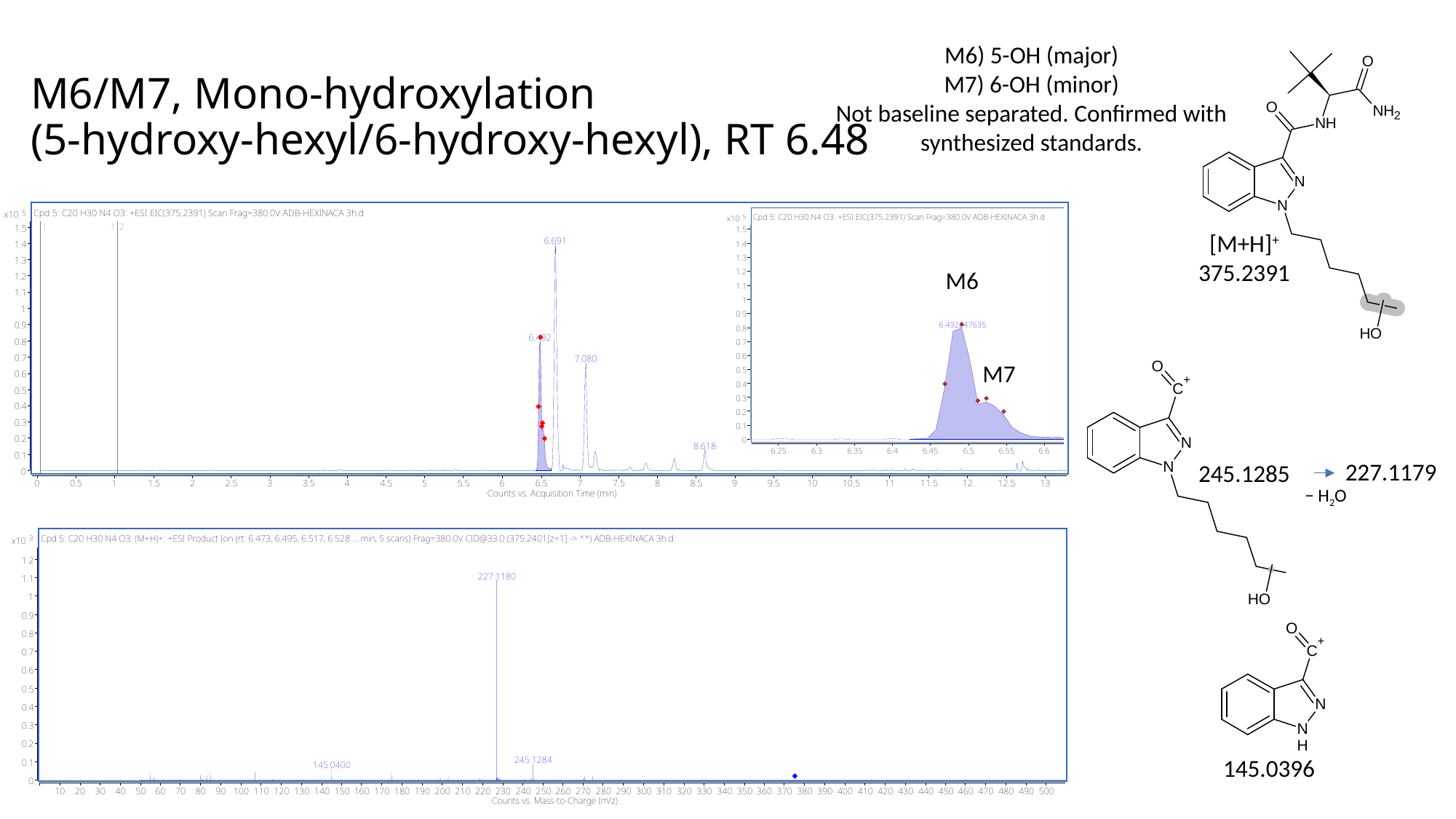

M6) 5-OH (major)
M7) 6-OH (minor)
Not baseline separated. Confirmed with synthesized standards.
[M+H]+
375.2391
227.1179
245.1285
− H2O
145.0396
# M6/M7, Mono-hydroxylation (5-hydroxy-hexyl/6-hydroxy-hexyl), RT 6.48
M6
M7

## Slide 9
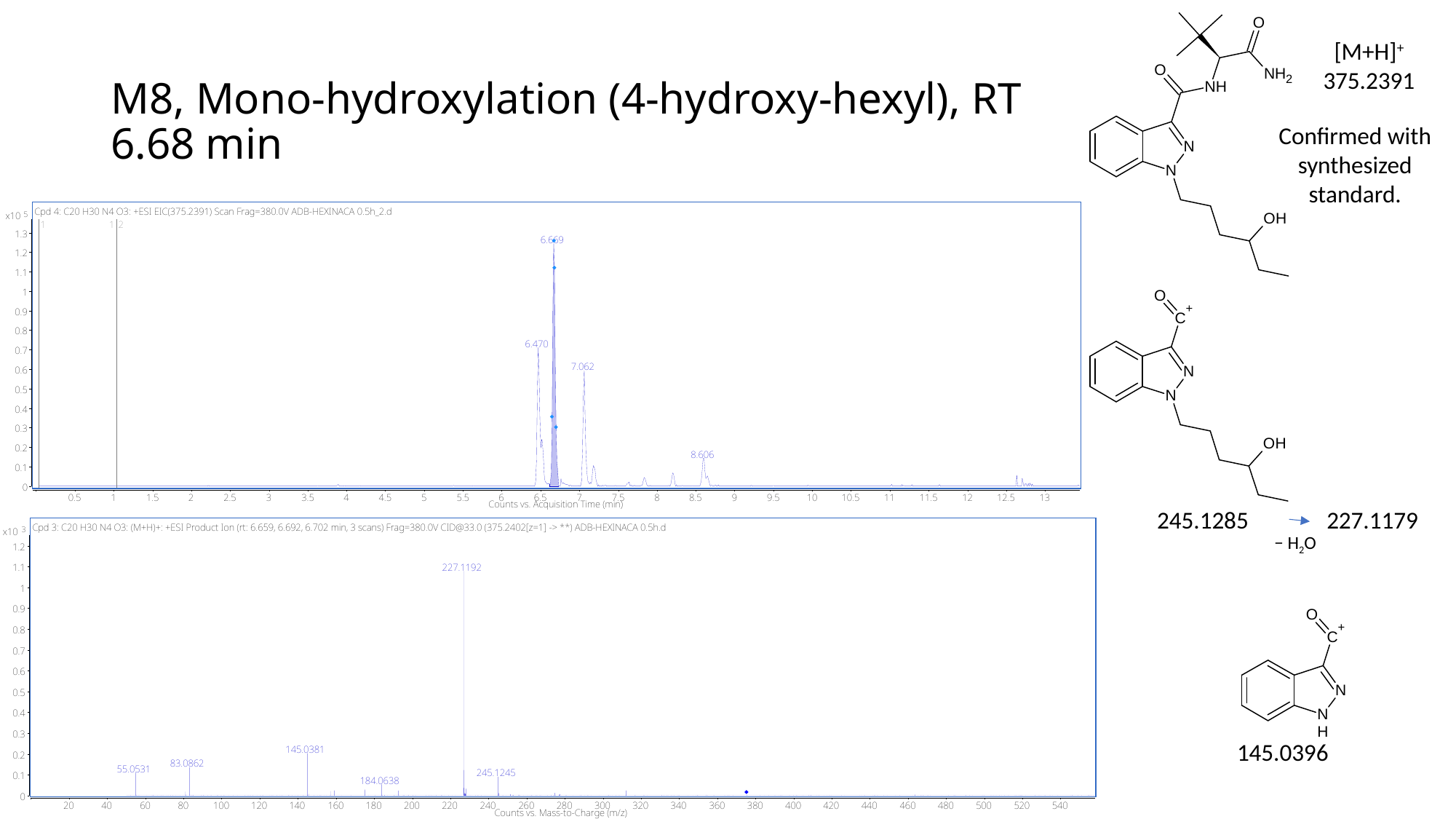

[M+H]+
375.2391
Confirmed with synthesized standard.
245.1285
227.1179
− H2O
145.0396
# M8, Mono-hydroxylation (4-hydroxy-hexyl), RT 6.68 min

## Slide 10
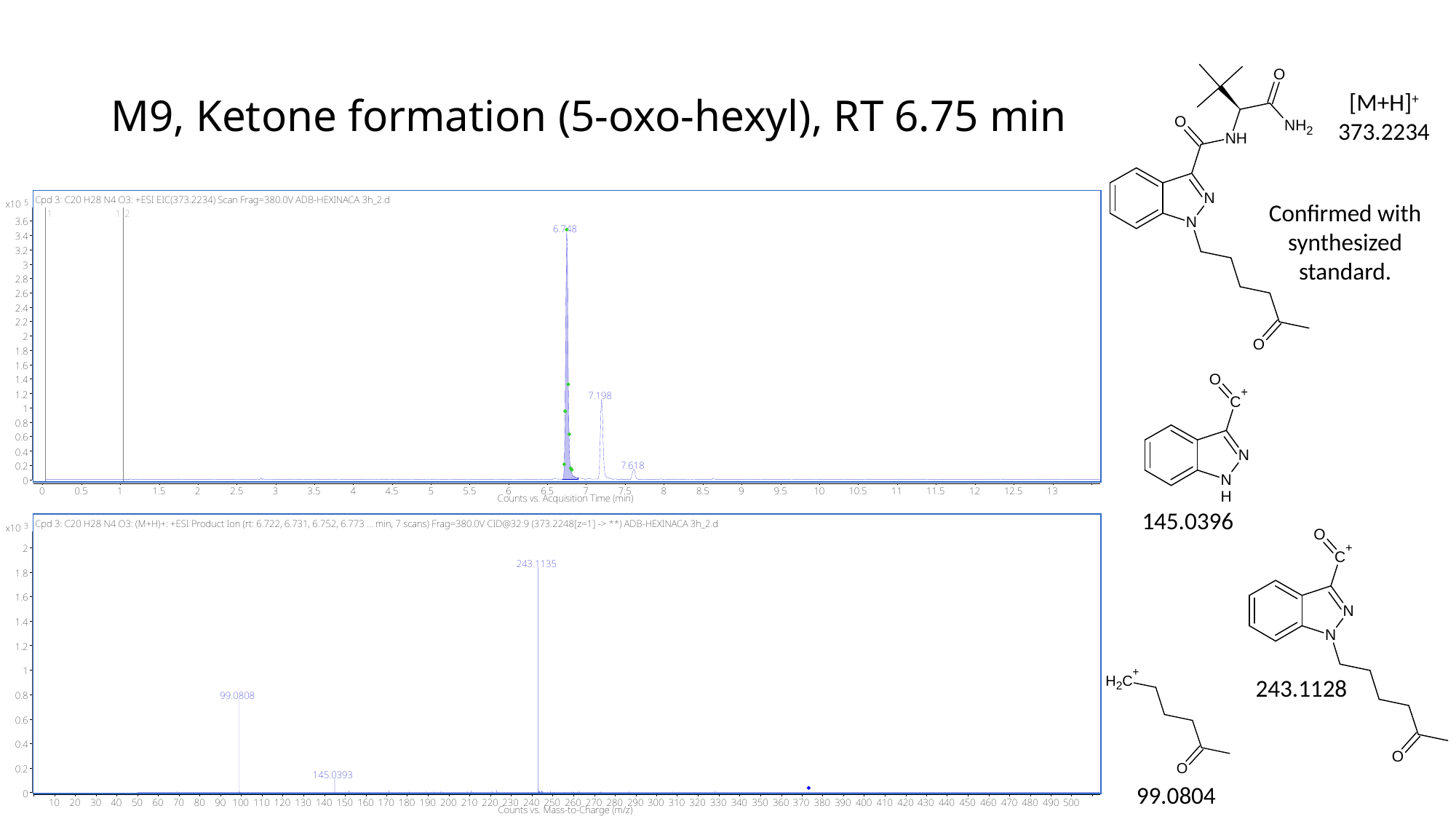

# M9, Ketone formation (5-oxo-hexyl), RT 6.75 min
[M+H]+
373.2234
Confirmed with synthesized standard.
145.0396
243.1128
99.0804

## Slide 11
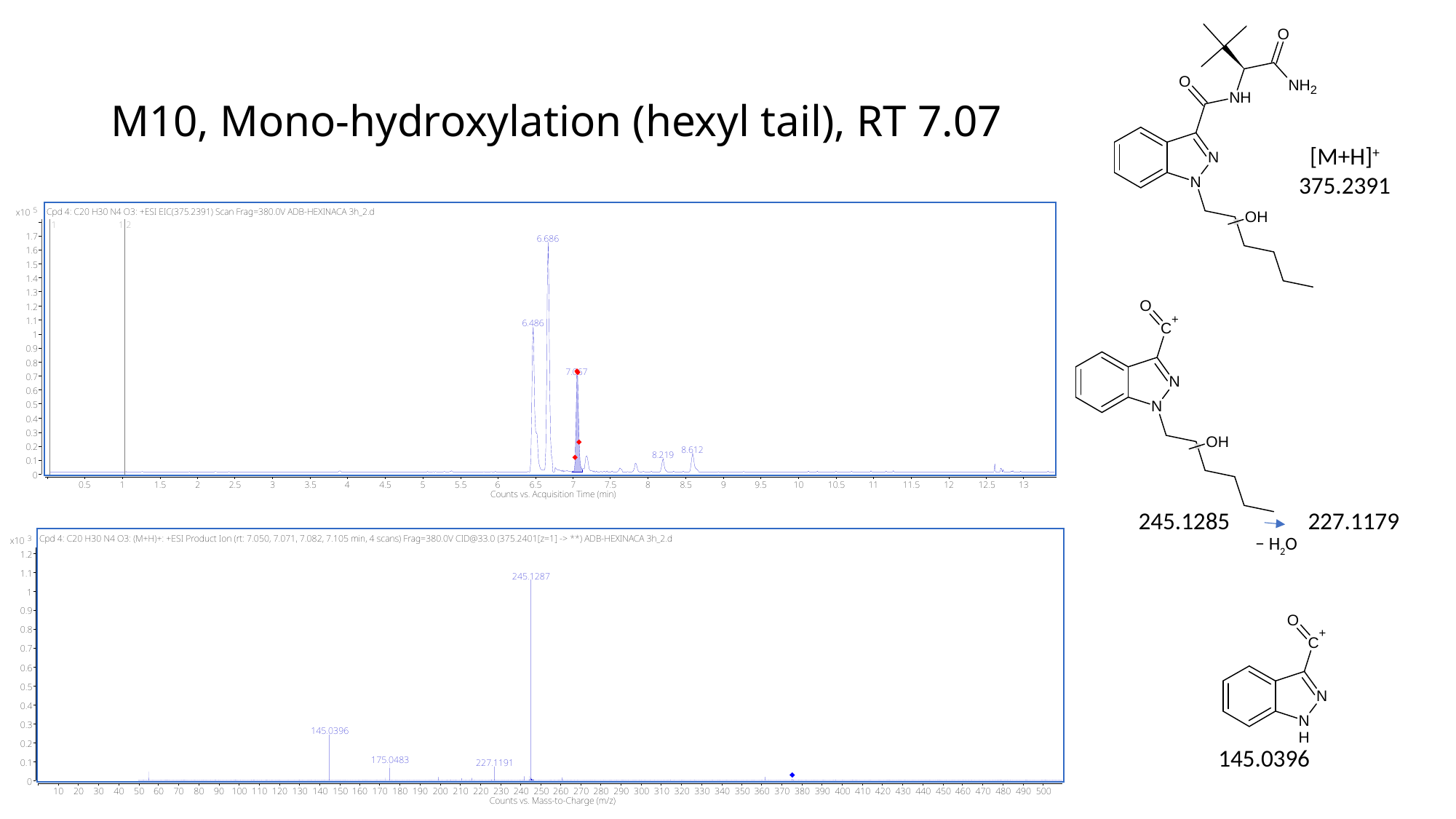

[M+H]+
375.2391
245.1285
227.1179
− H2O
145.0396
# M10, Mono-hydroxylation (hexyl tail), RT 7.07

## Slide 12
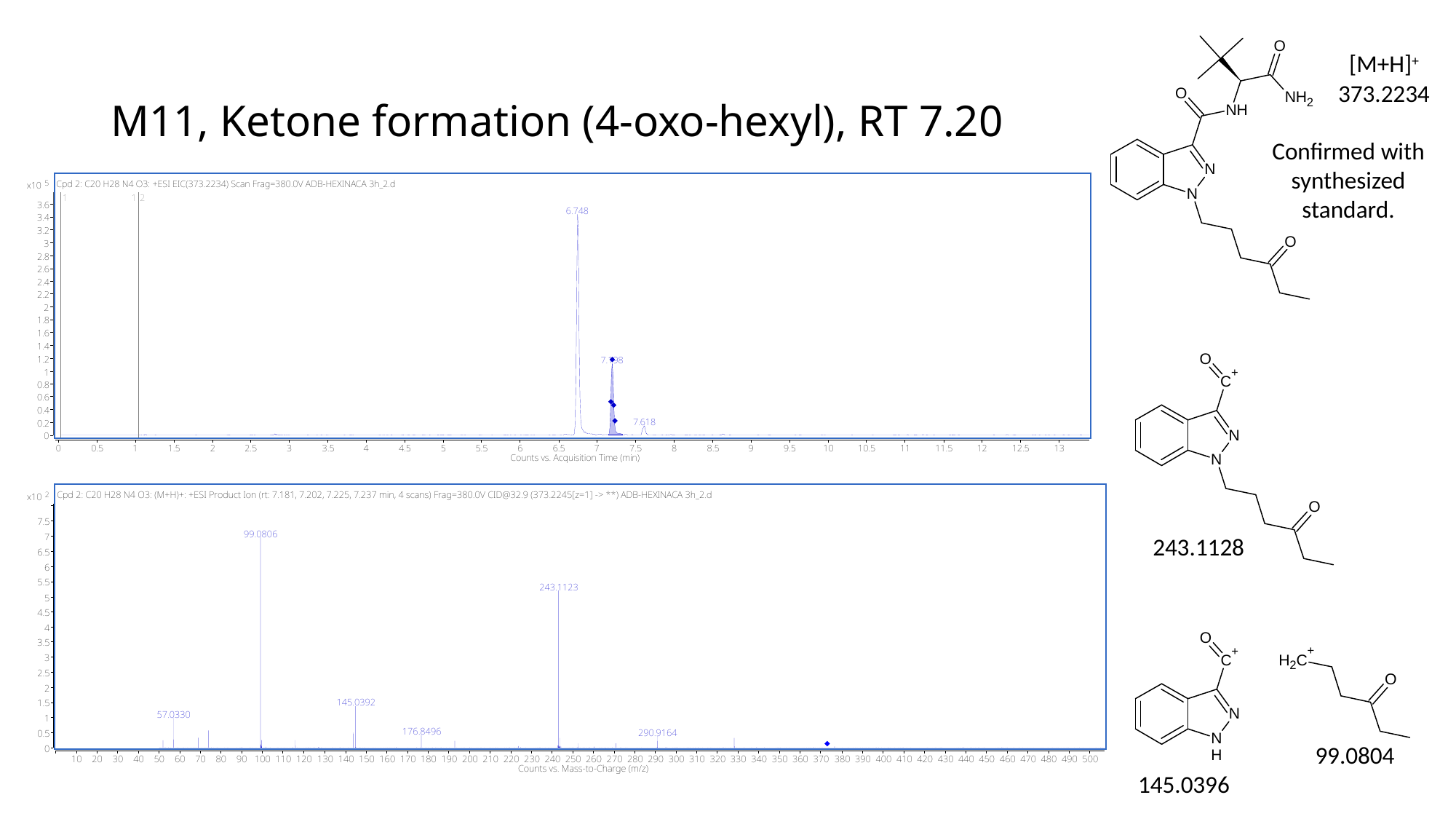

[M+H]+
373.2234
Confirmed with synthesized standard.
243.1128
99.0804
145.0396
# M11, Ketone formation (4-oxo-hexyl), RT 7.20

## Slide 13
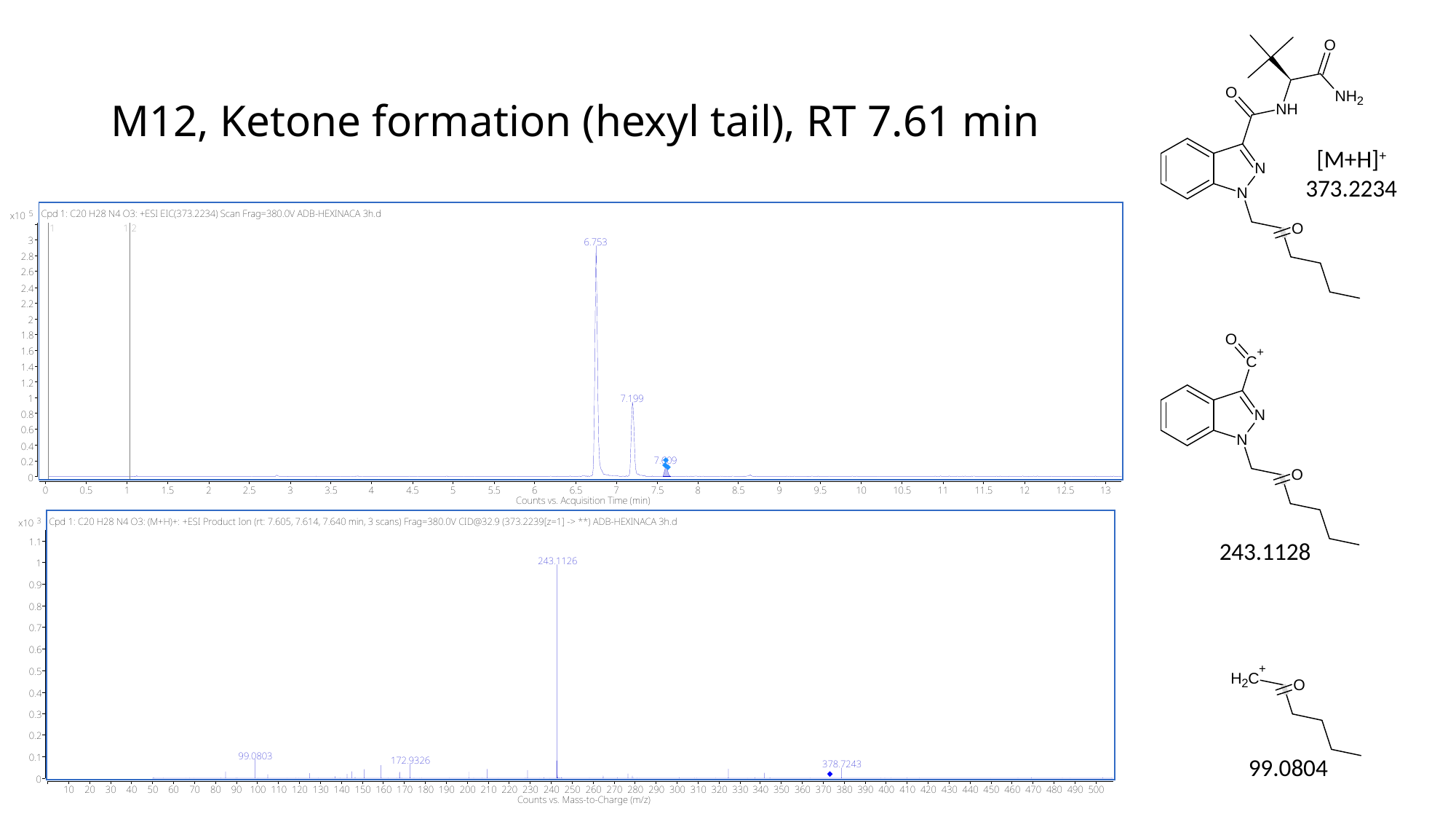

[M+H]+
373.2234
243.1128
99.0804
# M12, Ketone formation (hexyl tail), RT 7.61 min

## Slide 14
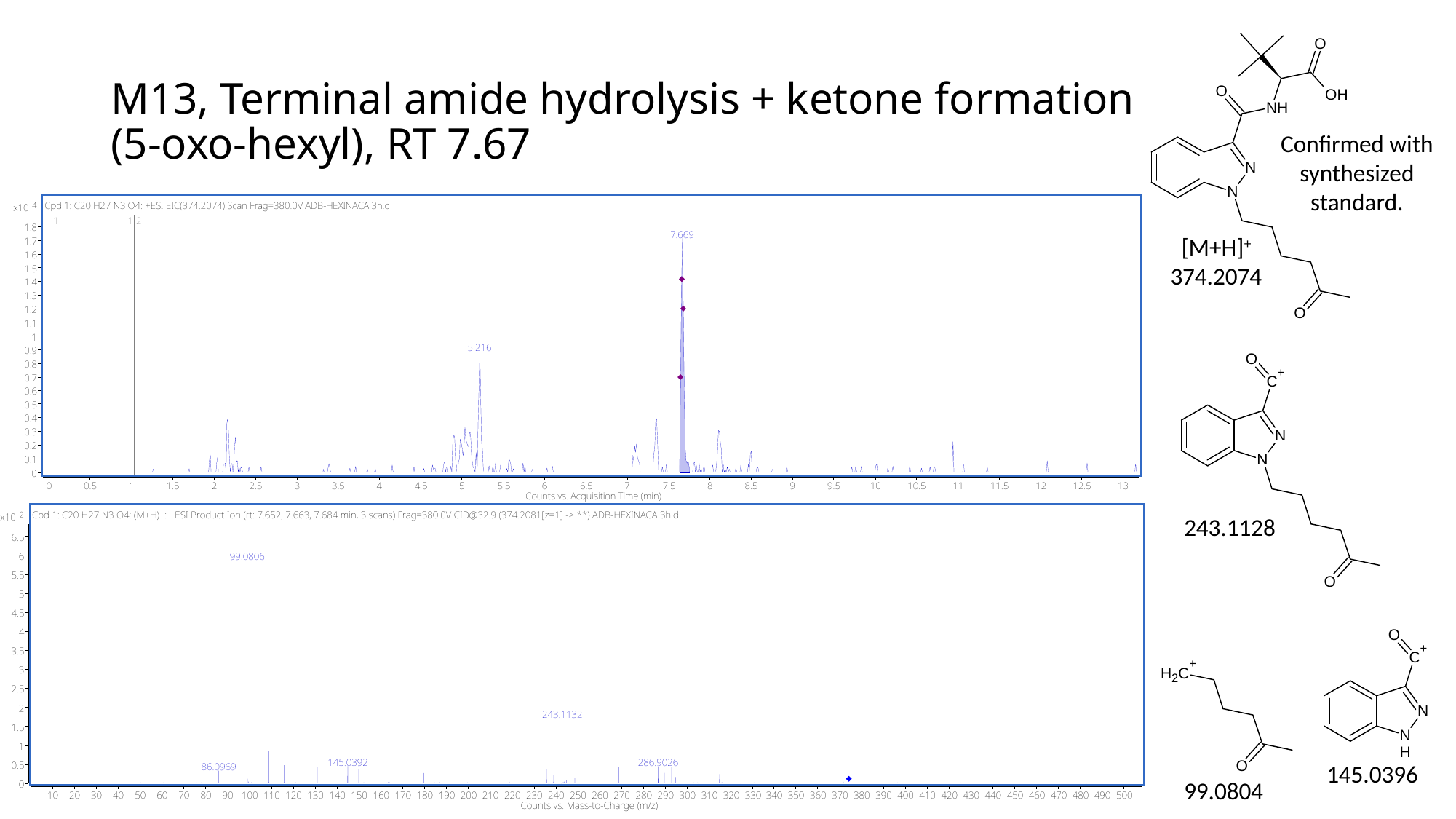

Confirmed with synthesized standard.
[M+H]+
374.2074
243.1128
145.0396
99.0804
# M13, Terminal amide hydrolysis + ketone formation (5-oxo-hexyl), RT 7.67

## Slide 15
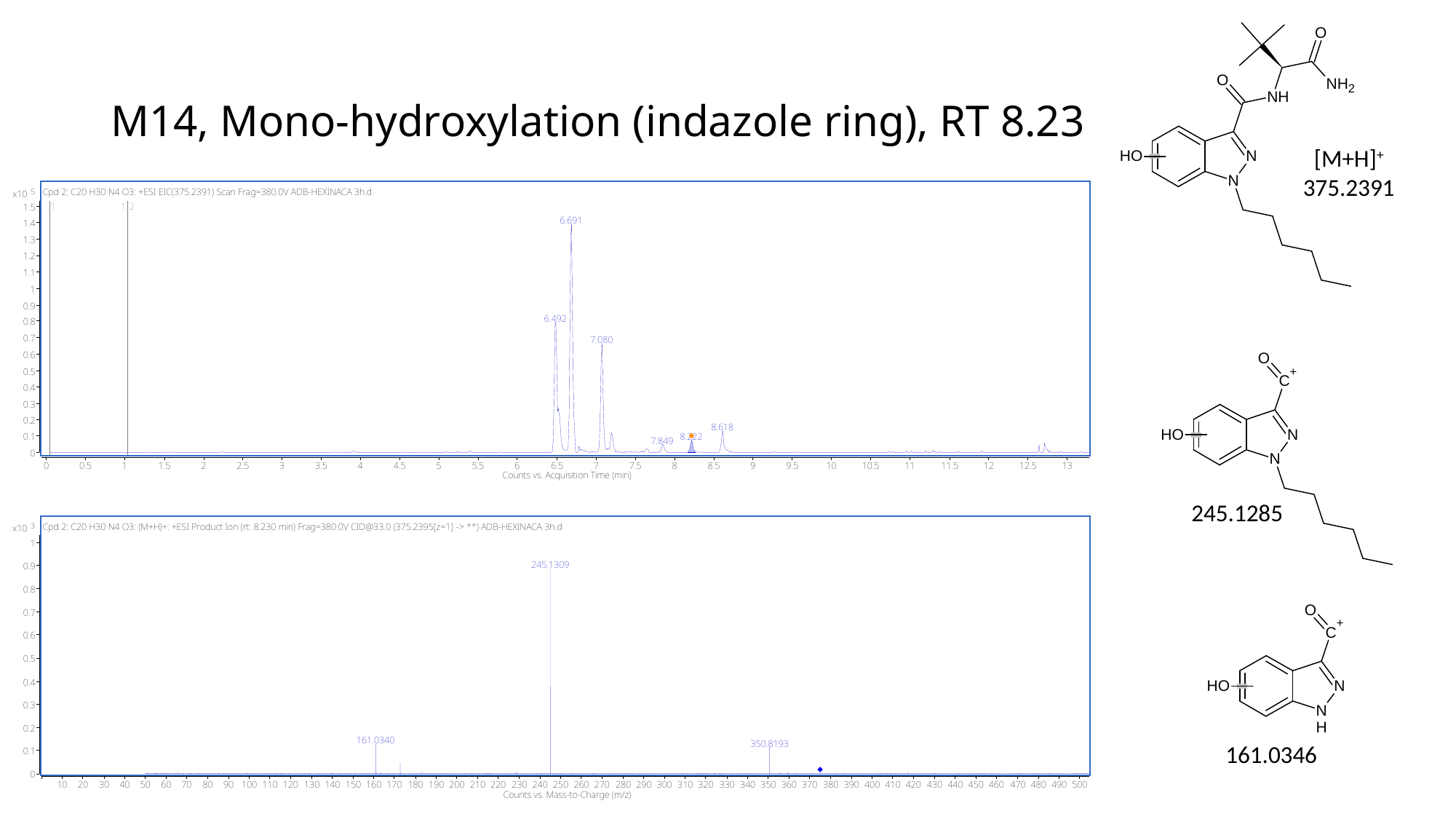

[M+H]+
375.2391
245.1285
161.0346
# M14, Mono-hydroxylation (indazole ring), RT 8.23

## Slide 16
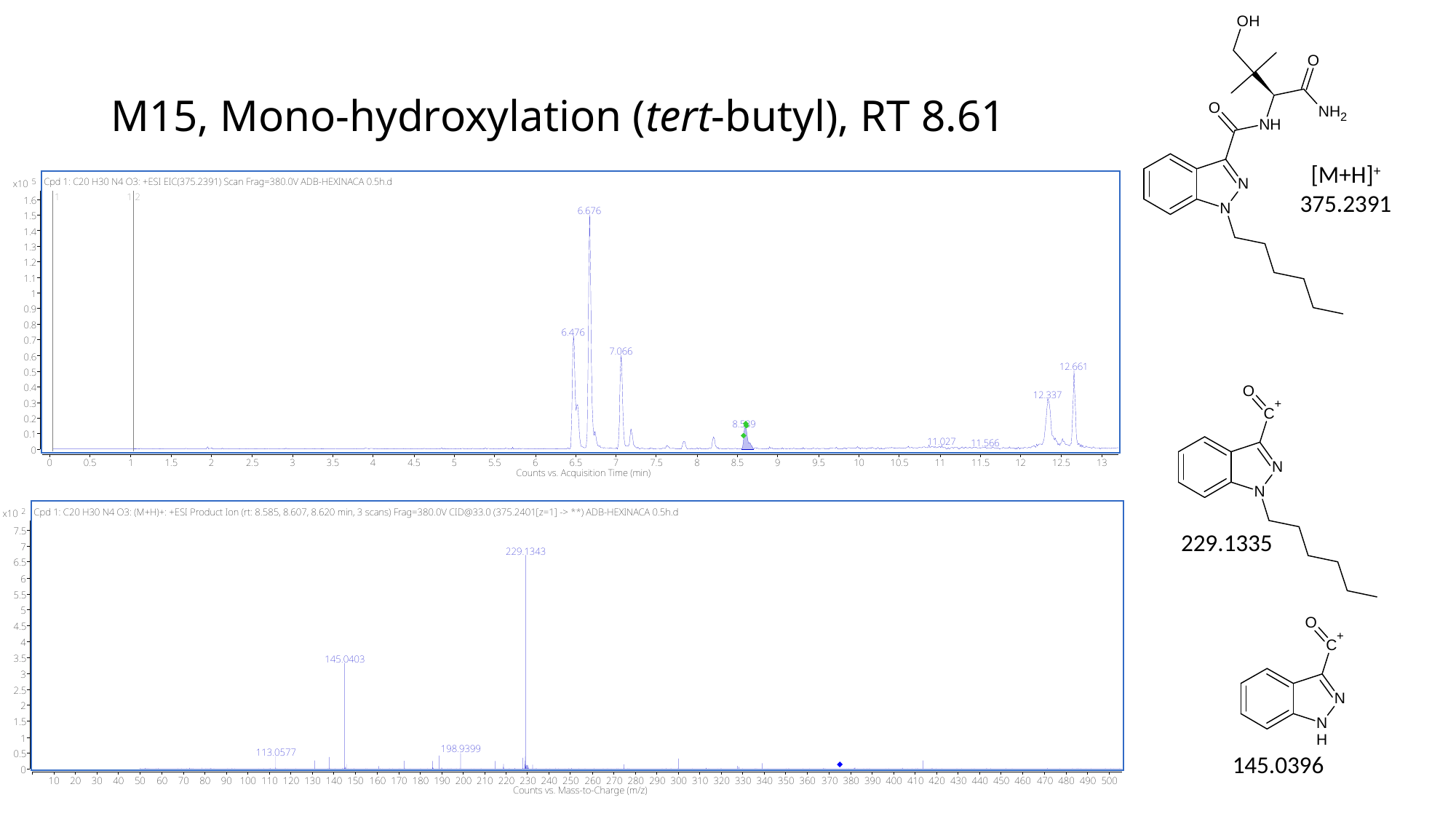

[M+H]+
375.2391
229.1335
145.0396
# M15, Mono-hydroxylation (tert-butyl), RT 8.61

## Slide 17
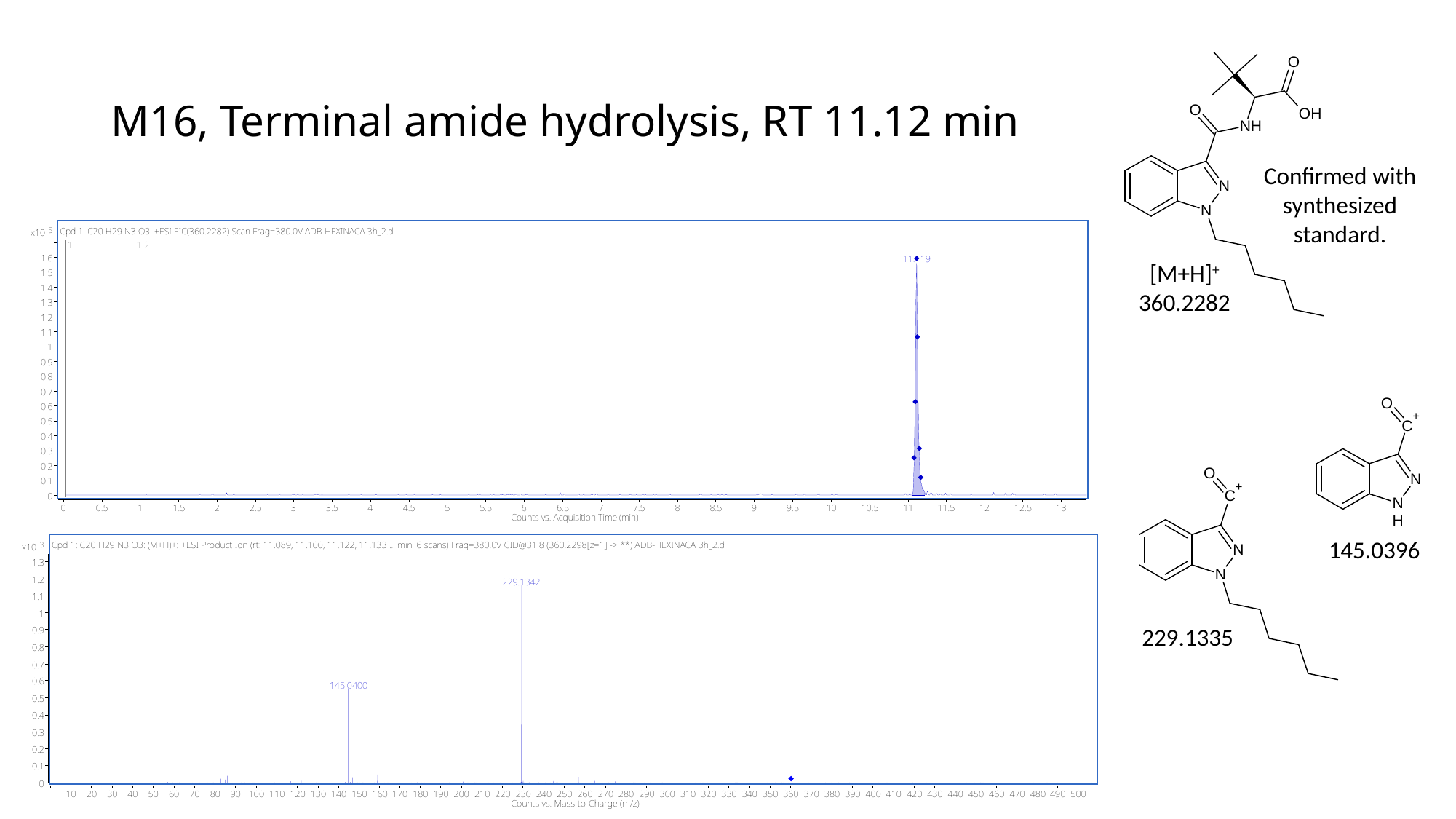

# M16, Terminal amide hydrolysis, RT 11.12 min
Confirmed with synthesized standard.
[M+H]+
360.2282
145.0396
229.1335
